# Evolution of vertebrate nicotinic acetylcholine receptors

**DOI:** 10.1186/s12862-018-1341-8

**Published:** 2019-01-30

**Authors:** Julia E. Pedersen, Christina A. Bergqvist, Dan Larhammar

**Affiliations:** Department of Neuroscience, Unit of Pharmacology, Science for Life Laboratory, Uppsala University, Box 593, SE-751 24 Uppsala, Sweden

**Keywords:** Acetylcholine, Receptor, Nicotinic, Gene duplication, Tetraploidization, Synteny, Paralogon, Ohnolog, Zebrafish, Spotted gar

## Abstract

**Background:**

Many physiological processes are influenced by nicotinic acetylcholine receptors (nAChR), ranging from neuromuscular and parasympathetic signaling to modulation of the reward system and long-term memory. Due to the complexity of the nAChR family and variable evolutionary rates among its members, their evolution in vertebrates has been difficult to resolve. In order to understand how and when the nAChR genes arose, we have used a broad approach of analyses combining sequence-based phylogeny, chromosomal synteny and intron positions.

**Results:**

Our analyses suggest that there were ten subunit genes present in the vertebrate predecessor. The two basal vertebrate tetraploidizations (1R and 2R) then expanded this set to 19 genes. Three of these have been lost in mammals, resulting in 16 members today. None of the ten ancestral genes have kept all four copies after 2R. Following 2R, two of the ancestral genes became triplicates, five of them became pairs, and three seem to have remained single genes. One triplet consists of *CHRNA7*, *CHRNA8* and the previously undescribed *CHRNA11*, of which the two latter have been lost in mammals but are still present in lizards and ray-finned fishes. The other triplet consists of *CHRNB2*, *CHRNB4* and *CHRNB5*, the latter of which has also been lost in mammals. In ray-finned fish the neuromuscular subunit gene *CHRNB1* underwent a local gene duplication generating *CHRNB1.2*. The third tetraploidization in the predecessor of teleosts (3R) expanded the repertoire to a total of 31 genes, of which 27 remain in zebrafish. These evolutionary relationships are supported by the exon-intron organization of the genes.

**Conclusion:**

The tetraploidizations explain all gene duplication events in vertebrates except two. This indicates that the genome doublings have had a substantial impact on the complexity of this gene family leading to a very large number of members that have existed for hundreds of millions of years.

**Electronic supplementary material:**

The online version of this article (10.1186/s12862-018-1341-8) contains supplementary material, which is available to authorized users.

## Background

The combination of pharmacological and genetic studies has revealed a multitude of receptors for acetylcholine (ACh) in vertebrates. They belong to the superfamily of cysteine-loop ion channels named so after two extracellular cysteine residues forming a disulfide bond. This superfamily also includes receptors for 5-hydroxytryptamine (5-HT), GABA and glycine in vertebrates. In invertebrates also glutamate and histamine can serve as endogenous agonists, see [1]. The origin of the Cys-loop superfamily predates the emergence of eukaryotes. However, despite their name the Cys-loop is not absolutely conserved in all members of the superfamily [2], whereas a proline in this region is invariant leading the authors to suggest that the superfamily should be called the Pro-loop family. Some of the ACh channels are activated by nicotine, and although nicotine has very low affinity for several of the channels, the whole family is described as nicotinic ACh receptors, abbreviated nAChR.

The Cys-loop receptors are pentamers where each subunit consists of approximately 550 amino acid residues and has four transmembrane (TM1–4) regions. Both the amino-terminus and the carboxy-terminus are on the extracellular side which contains a signal peptide at the amino-terminus and several consensus sites for N-linked glycosylation in the extracellular parts. The TM2 region contains charged and polar residues that line the central ion pore. The biological role of the ACh receptors is to allow Na^+^ ion influx. They are expressed both pre- and postsynaptically [3]. On the postsynaptic side, the ion influx leads to depolarization and thereby these nAChRs function as excitatory receptors. On the presynaptic side, the nAChRs serve more of a modulatory role, regulating the release of neurotransmitters.

The nAChR expressed in the neuromuscular junction (NMJ) was the first to be structurally characterized in detail. This receptor is formed by two α subunits and one each of β, δ and ε. During embryonic development in mammals, the γ subunit is expressed but it is replaced by ε around birth. In rodents this switch takes place after the two first post-natal weeks [4]. The NMJ channel subunits when viewed from the extracellular space appear clockwise in the order α-δ-β-α-ε. Binding sites for ACh appear at the α-δ and α-ε interfaces. In the brain, the receptors are either heteropentamers consisting of two or three types of subunits, enabling great receptor combination diversity (e.g., two α4 and three β2, or two α6, two β2 and one β3, or α7β2) or homopentamers (α7) [5–7]. For these receptors ACh binding takes place at α-β and α-α interfaces, respectively. When a subunit does not directly participate in formation of the binding site, it is referred to as an accessory subunit, a position which can be occupied by the α3-α5 and β2-β4 subunits and it is the only position that the α5 and β3 may occupy in the pentamer [8].

Presently 16 genes for nAChR subunits are known in mammals including humans. The evolutionary relationships and origins of the vertebrate subunits have been difficult to resolve due to their variable evolutionary rates. Therefore other features have also been considered such as intron positions in the genes. A study by Le Novère and Changeux [9] of the 14 human genes that were known at the time proposed an evolutionary scheme with serial duplications occurring at different time points before and during vertebrate evolution. An analysis of the whole superfamily of Cys-loop channels using mostly mammalian sequences and a few from chicken, goldfish, *Xenopus laevis* and the ray *Torpedo marmorata* also suggested several serial duplications but did not calibrate the duplications to divergence time points of different evolutionary taxa [10]. Unsurprisingly, considering the limited amount of information, the two studies came to rather different conclusions. Subsequent analyses have resulted in yet additional variations of tree topologies [11–13]. Especially the evolutionary relationship of the NMJ-α subunit gene *CHRNA1* has been difficult to resolve, sometimes grouping with the other NMJ subunit genes, sometimes with the neuronal α-subunit genes.

During the 1990s data accumulated suggesting that vertebrate genomes had undergone two doublings, i.e. tetraploidizations, before the divergence of the major vertebrate lineages that subsequently became the classes recognized today. Based on sequence comparisons in combination with chromosomal locations of the genes, we proposed a duplication scheme that took into consideration the two tetraploidizations [14]. However, our data were almost exclusively based on information for the human genome through the OMIM database, Online Mendelian Inheritance of Man. Some resistance to the idea of two basal vertebrate tetraploidizations remained until two seminal reports described extensive evidence for quartets of related chromosome blocks, thus reflecting two tetraploidizations [15, 16]. In the meantime, strong evidence had been published for a teleost-specific tetraploidization [17] after it diverged from Sarcopterygii (tetrapods, lungfishes and coelacanths). The two basal vertebrate tetraploidizations are usually called 1R and 2R for the first and second round of whole genome duplication (WGD). The tetraploidization at the origin of the teleost lineage is called 3R.

With the availability of high-quality genome assemblies for several important vertebrate species, it is now possible to investigate the nAChR family with a broad approach that takes into consideration both amino acid sequences, intron positions, and the chromosomal locations of the genes in each species. The latter criterion has turned out to be useful to distinguish orthologs (species homologues) and paralogs (gene duplicates). For gene duplicates that arose in the 1R and 2R events (as well as other tetraploidizations), the term ohnologs is used in honor of Susumu Ohno who proposed tetraploidizations as a mechanism that generated additional genes in vertebrates [18].

We present here a comprehensive evolutionary analysis of the nAChR subunit genes in vertebrates with special focus on their relationships to the two basal vertebrate tetraploidizations and the teleost tetraploidization. We conclude that the 1R, 2R and 3R events together account for all nAChR gene duplications in vertebrates except two, showing that the genome doublings have had a large impact on the complexity of this gene family.

## Results

### Phylogenetic analysis shows extensive nAChR duplications in the time range of 1R and 2R

**Fig. 1:**
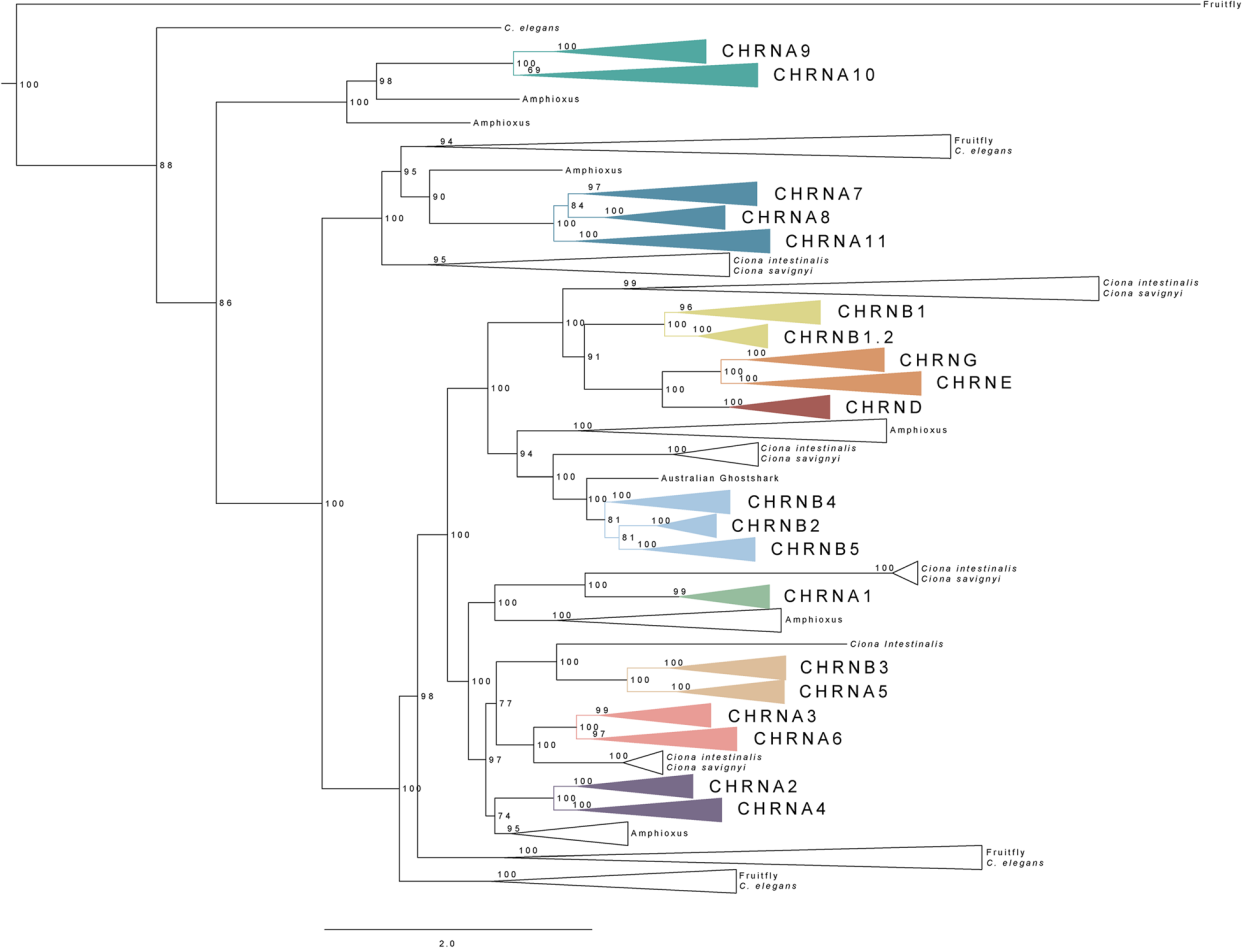
Phylogenetic maximum likelihood tree of the nAChR genes, rooted with the human *5HTR3A* and *5HTR3B;* the root is not displayed. The collapsed nodes represent the orthologs of the respective nAChR genes, where each color code corresponds to one nAChR gene subfamily. The invertebrate chordate (represented by amphioxus and tunicates) and protostome (represented by *C. elegans* and fruitfly) closest homologs are shown as non-colored nodes. The tree topology is supported by a non-parametric Ultra-Fast Bootstrap (UFBoot) analysis

The phylogenetic analysis of the nAChR genes from a broad selection of vertebrate species listed in Methods, rooted with the human 5HTR3A and 5HTR3B receptors, is shown in Fig. 1. Also the GABA-A family has been used as outgroup and gave the same topology (not shown). The tree shows that among the members of the nAChR family, the subfamily comprised of the *CHRNA9* and *CHRNA10* genes forms the earliest branch, in agreement with previous analyses [9, 10] but in contrast to a recent study [13]. This clade has two amphioxus sequences as closest relatives showing that the duplication of the *CHRNA9*/*CHRNA10* ancestor coincides with the time range of 1R and 2R, as confirmed by our paralogon analysis (see below). The *CHRNA9* and *CHRNA10* are present in all species included in this analysis, except that *CHRNA10* has not been identified in Australian ghostshark. Furthermore, 3R duplicates are retained in all teleost species (Additional file 1). However, the genes in this subfamily have evolved at quite different rates, for instance the *CHRNA10* gene in human, mouse and opossum has evolved faster than in the other vertebrates, see Additional file 1. The next branch point leads to the clade forming the *CHRNA7*/*CHRNA8*/*CHRNA11* gene subfamily, also closely related to a group of invertebrate chordates as well as protostomes, including both fruitfly and *C. elegans* (Fig. 1). Notably, there seems to have been a local expansion in *C. elegans*, resulting in nine genes (Additional file 1). The *CHRNA7* gene is present in all vertebrates investigated. The zebrafish and medaka genomes have retained a 3R duplicate of *CHRNA7*. The *CHRNA8* gene has not been identified in mammals, but it is present in chicken, lizard, frog, cartilaginous fish and ray-finned fish. A 3R duplicate of the *CHRNA8* gene is present in stickleback, medaka and fugu, but not in zebrafish (Additional file 1). Interestingly, during the analysis an additional subfamily member that has not been previously described was identified, here referred to as *CHRNA11*. It is present in lizard, coelacanth, cartilaginous fish and ray-finned fish and 3R duplicates are present in medaka, stickleback and fugu (Additional file 1). The 3R duplicates of *CHRNA11* differ considerable from each other in their evolutionary rates. Also *CHRNA11* in Australian ghostshark has diverged considerably.

The ancestor of the remainder of the nAChR family is also closely related to two groups of invertebrate sequences, where there have been extensive local duplications in *C. elegans* and fruitfly (Additional file 1). The ancestral deuterostome gene duplicated further giving rise to one ancestral gene for the four vertebrate subfamilies consisting of the CHRNB1/CHRNB1.2, CHRND, CHRNE/CHRNG and CHRNB2/CHRNB4/CHRNB5 genes, respectively (Fig. 1). The CHRNB2/CHRNB4/CHRNB5 clade has as sister group an amphioxus clade containing no less than nine genes, indicating an amphioxus-specific expansion. Subsequently, the olfactores clade underwent a duplication leading to the ancestors of the vertebrate clades comprised by the *CHRNB1/CHRNB1.2* and *CHRND/CHRNE/CHRNG* genes on the one hand, and the *CHRNB2/CHRNB4/CHRNB5* genes on the other. Each of these two clades has a sister group in tunicates (Fig. 1). Possibly, the duplication took place already in the chordate ancestor, whereupon one of the two clades was lost in amphioxus. Finally, additional duplications took place in the vertebrates. The ancestor of the *CHRNB2*/*CHRNB4*/*CHRNB5* subfamily was triplicated at a time point that coincides with 1R and 2R, supported by a group of tunicate sequences present basally to the subfamily (Fig. 1), as confirmed by our paralogon analysis (see below). The *CHRNB2* gene has not been identified in spotted gar or Australian ghostshark and the gene has not retained any 3R paralogs in teleosts (Additional file 1). The *CHRNB4* gene has not been identified in lizard nor coelacanth, but it is present in turtle. This gene has retained no 3R duplicates in teleosts. The *CHRNB5* gene has a different phylogenetic distribution, as it is found in Australian ghostshark, spotted gar and teleosts, and in the teleost lineage it has retained 3R duplicates in zebrafish, medaka and fugu.

The *CHRNB1* gene encodes a subunit of the NMJ receptor but it has not been found in chicken, lizard, frog or Australian ghostshark. As this subunit is an obligate member of the mammalian NMJ heteropentamer, the genomes of additional species genomes were screened in silico and *CHRNB1* was found to be present in other reptiles, such as turtle, python and American alligator. The *CHRNB1* gene seems to have no surviving duplicates from tetraploidization events. In spotted gar and teleosts, on the other hand, a local duplication of *CHRNB1* in the ancestor of ray-finned fishes gave rise to the *CHRNB1.2* gene (Fig. 1 and Additional file 1), located in close proximity to the *CHRNB1* gene on the same chromosome (see Fig. 7). Alternatively but less parsimoniously, the duplication could have occurred before the actinopterygian–sarcopterygian split, in which case it would have been followed by a loss of *CHRNB1.2* in the sarcopterygian lineage. The common ancestor of the NMJ subunit genes *CHRND*, *CHRNE* and *CHRNG* first underwent a local duplication, giving rise to the *CHRND* gene and the ancestor of *CHRNE* and *CHRNG*, then the 1R and 2R duplications resulted in the *CHRNE* and *CHRNG* genes (Fig. 1). The *CHRND* has not been found in stickleback, but is present in the rest of the species investigated. The *CHRNE* gene has not been found in chicken, frog or Australian ghostshark but it is present in python, turtle and American alligator. Both the *CHRNB1* and *CHRNE* genes are present in lizard and *CHRNE* in frog, possibly indicating that the genes exist also in the chicken genome but have not yet been sequenced. The *CHRNG* was also not identified in the Australian ghostshark, nor in medaka. Close inspection of the phylogenetic tree (see Additional file 1) shows quite varying evolutionary rates among tetrapod *CHRNE* sequences. Neither of the *CHRNB1*, *CHRNB1.2*, *CHRND*, *CHRNG* or *CHRNE* genes have retained additional teleost duplicates. Also, no close relatives of these genes were found in amphioxus but both of the tunicate species have a gene most closely related to the ancestor of these NMJ genes, and it has evolved very rapidly in the tunicate lineage (Fig. 1 and Additional file 1).

An ancestral CHRNA-like gene generated four copies, each one becoming the ancestor of one of the following subfamilies: *CHRNA1* (a single member), *CHRNA5*/*CHRNB3*, *CHRNA3*/*CHRNA6* and *CHRNA2*/*CHRNA4*. The most basal lineage of these four is the one formed by the *CHRNA1* gene. Despite its function as a subunit present in the NMJ receptors, our phylogenetic analyses show that the *CHRNA1* gene clusters together with the other α-subunit genes rather than the other four NMJ subunit genes (Fig. 1). The *CHRNA1* gene is present in all species investigated, and in contrast to the rest of the NMJ genes, it has retained 3R duplicates in stickleback, medaka and fugu. A local duplicate of *CHRNA1* is also present in frog, as previously described [19]. Two tunicates are found basal to *CHRNA1* and in common with the *CHRNB2*/*CHRNB4*/*CHRNB5* subfamily, the *CHRNA1*-like sequences in amphioxus contain a species-specific expansion with four genes present (Fig. 1 and Additional file 1).

The remaining three CHRNA-like genes were all duplicated during what seems to be the time period spanning 1R and 2R. The subfamily containing the *CHRNA5* and *CHRNB3* genes has one basal tunicate relative. The *CHRNA5* gene has not been found in lizard, but it is present in turtle. The *CHRNB3* is present in all species included in this analysis. The *CHRNB3* gene has retained the 3R duplicate in teleosts, in contrast to *CHRNA5* which retained no duplicate in any of the teleosts. The *CHRNA3* and *CHRNA6* genes form a separate subfamily, with a group of tunicate sequences as closest relatives. The *CHRNA3* gene has not been identified in lizard, but is present in turtle. The *CHRNA6* gene has not been found in coelacanth. There are 3R duplicates for *CHRNA6* in medaka, stickleback and fugu, but none for *CHRNA3*. In the *CHRNA2*/*CHRNA4* gene subfamily, finally, the *CHRNA2* and *CHRNA4* genes have not been identified in Australian ghostshark. A *CHRNA4* 3R duplicate is present in zebrafish. The *CHRNA2* gene has retained a duplicate both in stickleback and zebrafish. There are two close relatives in amphioxus to the *CHRNA2*/*CHRNA4* subfamily.

Taken together, the phylogenetic analyses show that the nAChR family can be divided into 10 subfamilies, each of which had one ancestral gene before the origin of the vertebrates and the two vertebrate tetraploidizations. Eight of these 10 ancestral genes seem to have orthologs in either tunicates or amphioxus or both. The ancestor of the clade consisting of *CHRNB1/CHRNB.2*, *CHRND*, and *CHRNE-CHRNG* appears to have triplicated after the tunicate lineage branched off, but before 1R, and subsequently 1R/2R generated the two additional duplicates shown in Fig. 7 (resulting in the *CHRNB1/CHRNB1.2* pair and the *CHRNE/CHRNG* pair). The analyses show that the 10 ancestral subfamily genes expanded in 2R, resulting in the 19 subunit genes present today in vertebrates. Furthermore, the timing of nAChR gene duplications resulting in the additional genes present in the teleosts coincides with the teleost specific tetraploidization 3R, except for *CHRNB1.2* that arose as a local duplicate of *CHRNB1* basally in the ray-finned fish lineage and the local *CHRNA1* duplicate in frog. Information about the nAChR sequences included in the analysis is provided in Additional file 2 and the multiple sequence alignment file is provided in Additional file 3. Additional alignment algorithms were tested for their possible advantage on the dataset as well as for control (these algorithms were: CLUSTAL and PRANK, data not shown), however MUSCLE was found to be most optimal for this dataset.

### Exon-intron organization differs among nAChR subunit genes

**Fig. 2:**
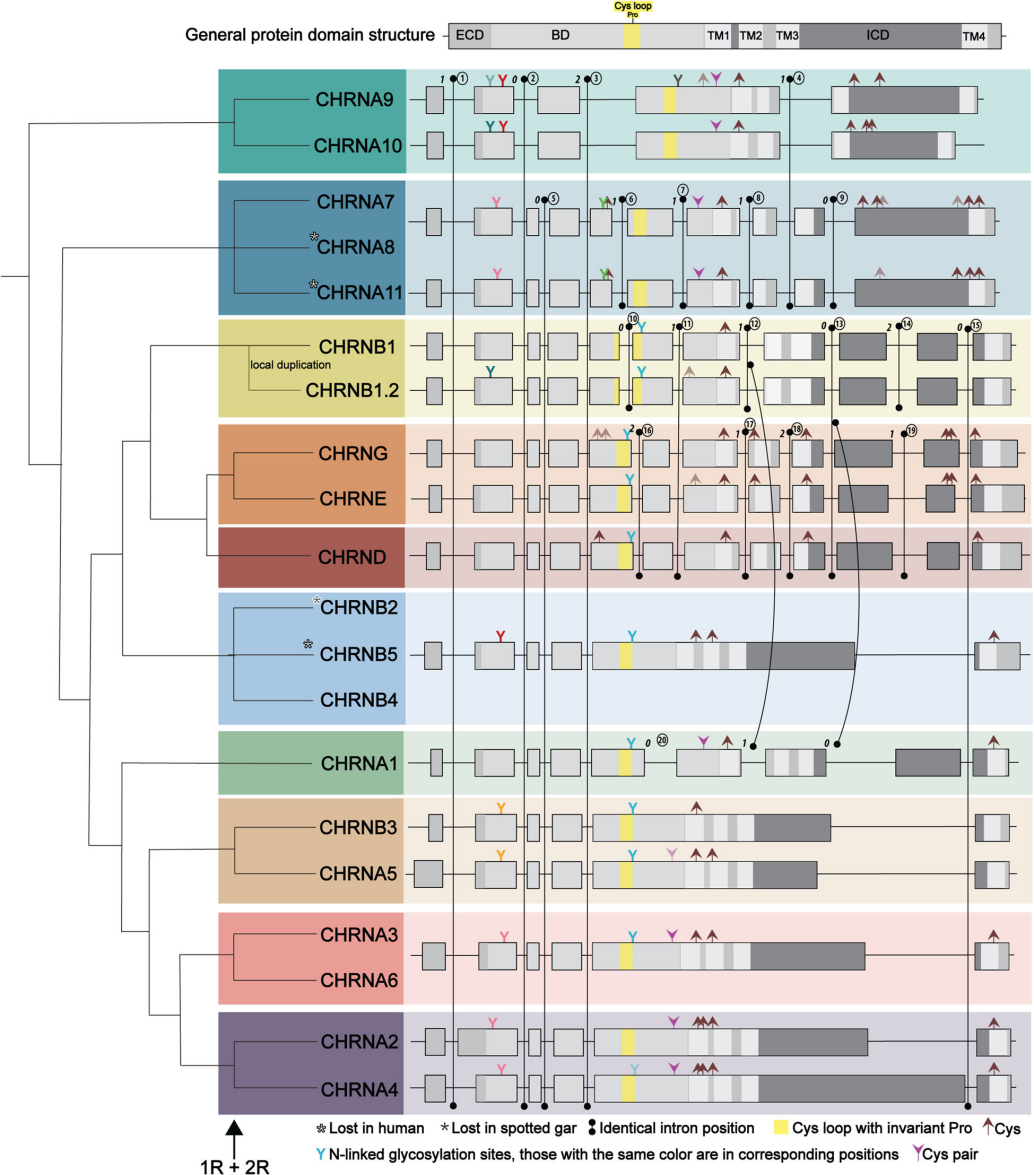
The left panel shows a phylogenetic tree based on intron insertions into the vertebrate sequences. The top panel shows a schematic outline of the general protein domain structure, including the extracellular domain (ECD), the binding domain (BD), the transmembrane regions 1–3 (TM1-TM3), the large intracellular domain (ICD) and finally the last TM4 region and the short extracellular carboxy-terminus. The Cys-loop with its conserved proline is marked in yellow. Below follows the exon-intron organization for each gene, if the organization is the same in two or more genes only one of them is shown. The color coding of the exons corresponds to the schematic outline in the top panel. The exons are drawn to scale and the splice phase is indicated at the beginning of each intron. Common intron positions are marked with a line, combining the positions that are identical between the different genes. The introns are numbered (1–20). The curved line indicates a common position shared between the *CHRNB1/CHRNB1.2* genes and the *CHRNA1* gene. Positions of cysteines, N-linked glycosylation sites (those with the same color are in corresponding positions) and the cysteine pair are also marked for each gene. Faded color means that it is not present in all orthologs

In addition to the sequence-based phylogenetic analysis, an intron-based phylogenetic analysis was carried out, i.e., a phylogenetic tree was created based on intron insertions into the vertebrate nAChR genes, similar to a previous analysis [9]. Figure 2 shows the exon-intron organization of the 19 vertebrate nAChR genes, with numbered introns, together with important protein features, namely positions of N-linked glycosylation sites, cysteine-pairs and single cysteines. This analysis displays all cysteine sites, although the ones present in the TM regions are not expected to have any particular role. The most parsimonious explanation for order of intron insertions into the nAChR genes is that the first three introns (intron 1–3, Fig. 2) were present in the common ancestor of all nAChR vertebrate genes, since these introns are shared between all genes. After *CHRNA9*/*CHRNA10* had branched off from the ancestor of the rest of the genes, an intron (intron 5, Fig. 2) was inserted dividing exon 3 into two. Subsequently, after the ancestor of the *CHRNA7*/*CHRNA8*/*CHRNA11* subfamily had branched off, the ancestor of the remainder of the genes received an intron (intron 15) before the region encoding TM4 (Fig. 2). Apart from these six introns (intron 1–5 and 15, Fig. 2) that are shared between several nAChR subfamilies, 14 additional introns have been gained specifically to subsets of subfamilies.

The exon-intron organization analysis divides the nAChR family into four groups. First comes the *CHRNA9*/*CHRNA10* subfamily, which as already mentioned has three introns (intron 1–3, Fig. 2) in common with all nAChR genes. In addition it has a fourth intron (intron 4, Fig. 2) which was gained independently in the ancestors of *CHRNA9*/*CHRNA10* and *CHRNA7*/*CHRNA8*/*CHRNA11*, respectively since it is present in all these genes. Alternatively, this intron was lost in the ancestor of all the other nAChR genes. The *CHRNA9*/*CHRNA10* group of genes contains the lowest number of introns, four in total (intron 1–4, Fig. 2). When comparing N-linked glycosylation sites and cysteines some differences between the *CHRNA9* and *CHRNA10* genes are observed, for instance there is one glycosylation site located just after the Cys-loop encoded by *CHRNA9* but not *CHRNA10* gene, whereas the glycosylation site encoded by the second exon is present only in some of the *CHRNA9* orthologs.

The second group based on exon-intron organization is formed by the *CHRNA7*/*CHRNA8*/*CHRNA11* subfamily which just as *CHRNA9*/*CHRNA10* has a quite distinct exon-intron organization. They contain nine introns (intron 1–9 in Fig. 2), four of which are unique to this subfamily (intron 6–9, Fig. 2). This is the only subfamily of genes where there is no glycosylation site present in any of the receptor subtypes close to the Cys-loop (Fig. 2). It seems that these genes (especially the *CHRNA7* and *CHRNA8*) have gained a higher number of cysteines in their last exon, which includes a part of the ICD as well as TM4.

The third group is formed by the NMJ subunit genes. The *CHRNB1*/*CHRNB1.2* genes share an identical exon-intron organization and they have two intron positions in common with *CHRND*/*CHRNE*/*CHRNG* (intron 11 and 13, Fig. 2), presumably inserted in their common ancestral gene. In addition, *CHRNB1*/*CHRNB1.2* share one intron (intron 12, Fig. 2) with *CHRNA1*. Further, the ancestral *CHRNB1*/*CHRNB1.2* gene received two additional introns (intron 10 and 14, Fig. 2). The positions encoding N-linked glycosylation sites and cysteines in the *CHRNB1* and *CHRNB1.2* sequences differ between the genes. In the second exon there is a glycosylation site encoded in *CHRNB1.2* which is not present in *CHRNB1*. Also, the *CHRNB1.2* gene codes for an extra cysteine. The genes in the *CHRND*/*CHRNE*/*CHRNG* clade have four unique introns (intron 16–19, Fig. 2), which were most likely gained in the *CHRND*/*CHRNE*/*CHRNG* ancestor. They all encode only one glycosylation site which is in close proximity to the Cys-loop. Additional differences are found for cysteine positions (Fig. 2). The remaining NMJ gene, *CHRNA1*, has a slightly different exon organization, containing fewer introns than the rest of the NMJ genes. As already mentioned, it shares one intron with *CHRNB1*/*CHRNB1.2* (intron 12, Fig. 2) and another one with *CHRNB1*/*CHRNB1.2* and *CHRND*/*CHRNE*/*CHRNG* (intron 13, Fig. 2). Intron 12 and intron 17 (Fig. 2) have the same splice phase although the intron position differs by one codon in *CHRND*/*CHRNE*/*CHRNG* relative to *CHRNB1*/*CHRNB1.2* and *CHRNA1*. Therefore the sequences of the *CHRND*/*CHRNE*/*CHRNG* genes were analyzed in detail to see if consensus splice donor-acceptor sites are present adjacently in the sequence, which could indicate that intron 12 and 17 are indeed the same intron insertion event that has subsequently undergone a one-codon shift by mutations. However, no obvious such possibility could be found, unless there have later been multiple substitutions that have eradicated any similarity, which would be a less parsimonious explanation. Hence, intron 17 in the *CHRND*/*CHRNE*/*CHRNG* clade could not be concluded to be the same as intron 12 in the *CHRNB1*/*CHRNB1.2* and the *CHRNA1* sequences. These similarities and dissimilarities in exon-intron organization results in two possible scenarios. Either the *CHRNA1* gene shared a common ancestor with the rest of the NMJ genes and therefore shares with these introns 12 and 13, whereupon it branched off and received intron number 20. Or, the common ancestor of *CHRNB1*/*CHRNB1.2* and *CHRNA1* may have received an intron at the same position independently and the *CHRND*/*CHRNE*/*CHRNG* ancestor may have received an intron one codon away, a scenario consistent with the clustering of *CHRNA1* with the neuronal α-genes in the sequence-based tree. What further differentiates the *CHRNA1* gene from the rest of the NMJ genes is the cysteine pair located in its sixth exon, which is characteristic for the α-subunits.

The fourth “intron-position clade” is the one that contains most of the gene family members, namely the *CHRNA2*-*CHRNA6*/*CHRNB2*–5 genes. They all share an organization that includes a very large exon 5, which distinguishes them from the rest of the nAChR genes. However, the length of the fifth exon as well as some features regarding locations of glycosylation sites, cysteines and the cysteine pair differ between the subunits. For instance, despite its position in the tree the *CHRNB3* gene lacks the cysteine-pair, just as the *CHRNB2*/*CHRNB5*/*CHRNB4* genes (Figs. 1 and 2). Also, not all of the *CHRNA5* orthologs contain the cysteine pair.

As described in the methods section the exon-intron comparison is based on the human and spotted gar sequences, and on one occasion the zebrafish (for *CHRNA11*). However, although the intron positions are quite well conserved in the rest of the species, there are some specific events associated with a few of the genes. For instance, some intron positions differ in opossum (in the *CHRNG* and *CHRNE* genes). However, it may be difficult to conclude whether these positions are true or whether they are results of artefacts in the genome assembly. When it comes to the teleosts, many of them have gained extra introns into the region encoding the ICD, the most variable part of the genes. Medaka, stickleback, fugu and zebrafish have all gained introns in the *CHRNA5* and *CHRNA9* genes and medaka, stickleback and fugu have gained introns in the *CHRNA1*, *CHRNA6*, *CHRNA10*, *CHRNB5* and *CHRNB3* genes. Further, stickleback has gained introns specifically in *CHRNA2*, *CHRNA4*, *CHRNB4* and *CHRNG* and zebrafish in the *CHRNA3* gene. The Australian ghostshark *CHRNB5* gene has also gained one intron. Finally, some introns seem to have been lost in medaka, stickleback and fugu for the *CHRNA8* gene and zebrafish lacks the first intron in the *CHRNA10* gene (Data not shown; available from the authors upon request).

### Synteny and paralogon analysis confirms expansion of the nAChR family following the vertebrate tetraploidizations

To test the phylogenetic results that indicate the existence of 10 ancestral (in the vertebrate predecessor) nAChR genes that expanded to 19 genes in the first vertebrate ancestor after 1R and 2R, the neighboring chromosomal regions of all nAChR genes were investigated to check for chromosome or block duplications consistent with 1R and 2R. Such related chromosome regions are said to belong to the same paralogon [20], i.e., a set of related chromosomal regions sharing members from the same gene families as a result of chromosome duplication. The 1R and 2R events together resulted in paralogons with four members (double tetraploidization) and the ensuing 3R event in teleosts gave paralogons with up to eight members. Investigation of the chromosomal positions for the nAChR genes showed that the members of each subfamily shared neighboring families, i.e., the neighboring genes also belong to subfamilies that have members on the same chromosomes as the nAChR subfamily, implying that they arose by block (chromosome) duplication. As the phylogenetic analyses (Fig. 1, Additional file 1) showed that the duplications occurred at the origin of the vertebrates, they all probably duplicated as a result of the 1R and 2R which took place in that time.

**Fig. 3:**
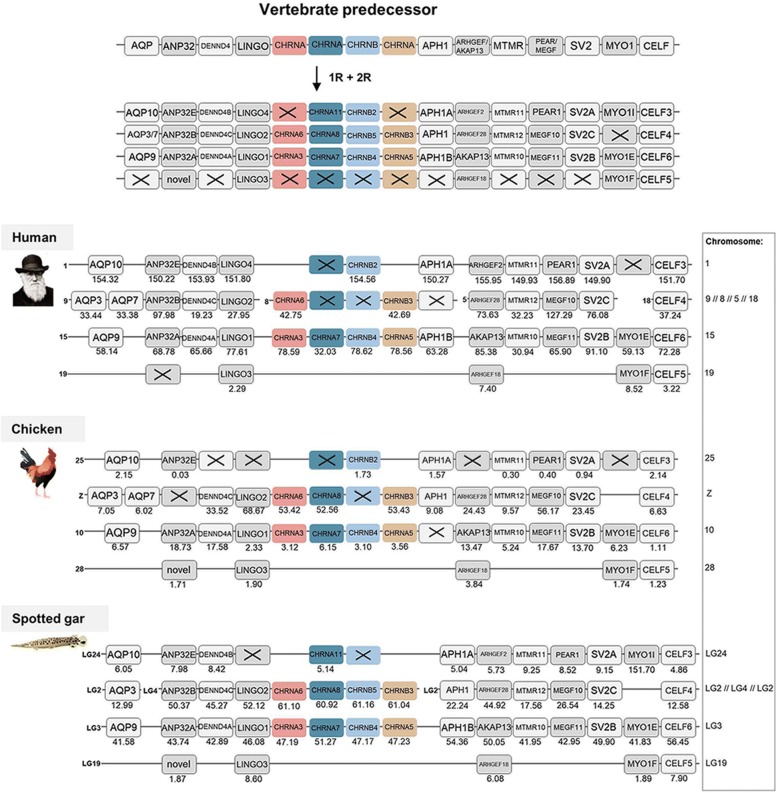
Analysis of the evolutionary history of the nAChR family with chromosomal locations of the nAChR genes and their neighboring gene families in human, chicken and spotted gar. Crosses indicate gene loss or gene not yet identified. Chicken and spotted gar illustrations are re-used with permission from Daniel Ocampo Daza, source: www.egosumdaniel.se and the human image is re-used with permission from https://commons.wikimedia.org. The duplication scheme and neighboring gene families for the *CHRNA6/CHRNA3, CHRNA11/CHRNA8/CHRNA7, CHRNB2/CHRNB5/CHRNB4* and *CHRNB3/CHRNA5* genes. The right panel summarizes chromosomes included. This paralogon is referred to as paralogon 1 in this study

The *CHRNA5*/*CHRNA3*/*CHRNB4* gene cluster is present on human chromosome 15, where the *CHRNA7* gene is also found. The phylogenetic analyses had indicated that the *CHRNB2*, *CHRNB5* and *CHRNB4* genes share a common ancestor, which had triplicated in 1R and 2R, or rather quadrupled after which one of the members was lost. The same scenario was indicated for the common ancestor of the *CHRNA7*, *CHRNA8* and *CHRNA11* genes. Following exclusion criteria stated in methods section, 11 gene families found in regions surrounding the nAChR genes just mentioned were analyzed and included, namely: *AQP, ANP32E, DENND4, LINGO, APH1, ARHGEF, MTMR, MEGF, SV2, MYO1* and *CELF* (Fig. 3). Based on the results of the phylogenetic analyses [21], the chromosomal positions for each member of these families were used to deduce a likely evolutionary scheme for human, chicken and spotted gar (Fig. 3). The similarity in the gene repertoires between the four chromosomal regions and species argues strongly that they arose as a result of quadruplication in 1R and 2R. Following a tetraploidization event, some duplicates may be lost, which is in fact a quite common scenario. Losses of genes, or genes that have not yet been identified, are indicated with crosses. Although the crosses are displayed on chromosomes, we cannot know the location of the gene at the time of the loss. However, four of the neighboring families (the *ANP32, LINGO, ARHGEF* and *CELF*) have retained all four copies, and they define four member chromosomes of this paralogon in the chicken genome. Although the rest of the neighboring gene families investigated have lost one and sometimes two members, those that are still present are located on the same four chromosomes as the gene families with full quartets, thus supporting the 1R and 2R events generating a paralogon. Three of the paralogon members in human are intact chromosomes and likewise for the spotted gar. As seen in the figure, the second chromosomal line in the human genome actually includes four different chromosomes (chromosome 9, 8, 5, 18), and the spotted gar orthologs are split into two different chromosomes (LG2 and LG4). This can be explained by translocations that have occurred after 1R and 2R, as the corresponding gene family members are located on one chromosome only in chicken (chromosome Z). Taken together, the chromosomal results from these species lead to the conclusion that these gene families all derive from an ancestral chromosome that was quadrupled in 1R and 2R and this paralogon has in a previous study been referred to as paralogon A [15]. In the present study we refer to it as paralogon 1 in relation to the nAChR gene family evolution.

**Fig. 4:**
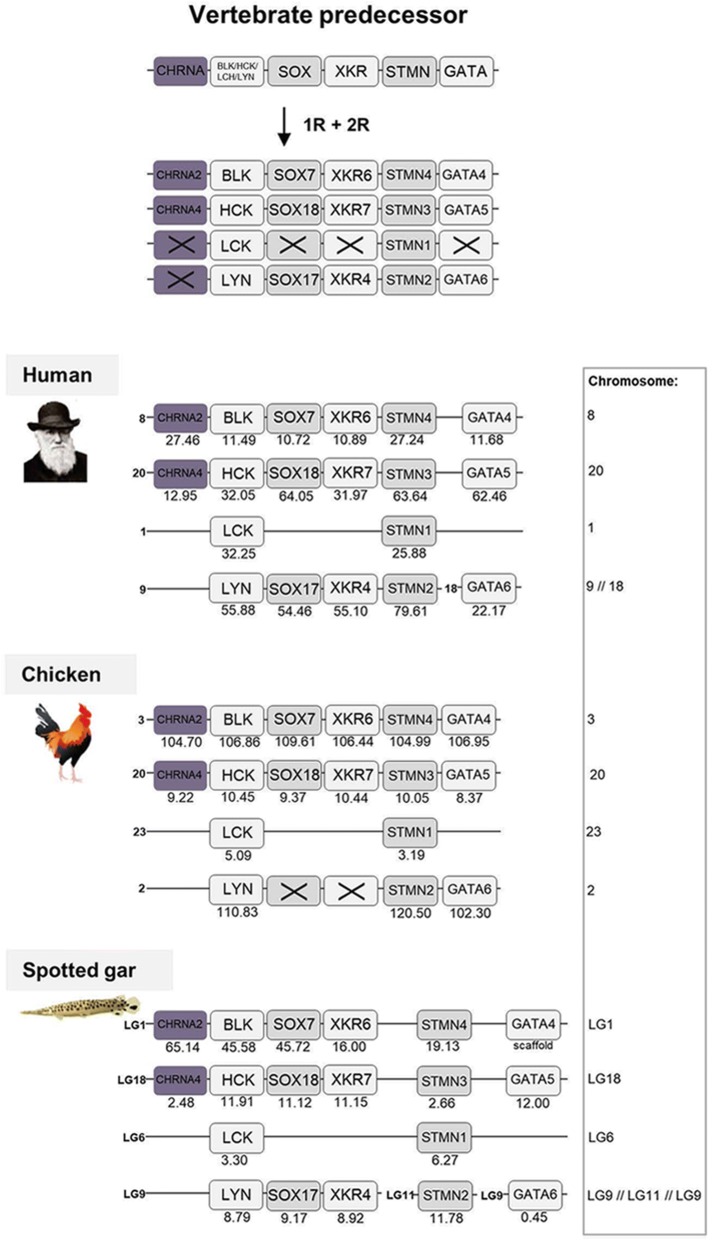
Analysis of the evolutionary history of the nAChR family with chromosomal locations of the nAChR genes and their neighboring gene families in human, chicken and spotted gar. Crosses indicate gene loss or gene not yet identified. Chicken and spotted gar illustrations are re-used with permission from Daniel Ocampo Daza, source: www.egosumdaniel.se and the human image is re-used with permission from https://commons.wikimedia.org. The duplication scheme and neighboring gene families for the *CHRNA2/CHRNA4* genes. This region of gene families have been analyzed in depth in previous studies from our lab, see Dreborg et al. (2008) and Cardoso et al. (2016). The right panel summarizes chromosomes included. This paralogon is referred to as paralogon 2 in this study

The *CHRNA2* and *CHRNA4* genes are located in separate regions compared to the other nAChR genes, on human chromosome 8 and 20, respectively. These regions and their neighboring gene families have been analyzed in depth in previous studies from our lab, where analyses showed that these regions belong to a paralogon that resulted from a 2R event [22, 23], namely paralogon B according to the classification provided in [15], which is also displayed in Fig. 4 as paralogon 2 in this study. Two neighboring gene families with complete quartets are included in Fig. 4.

**Fig. 5:**
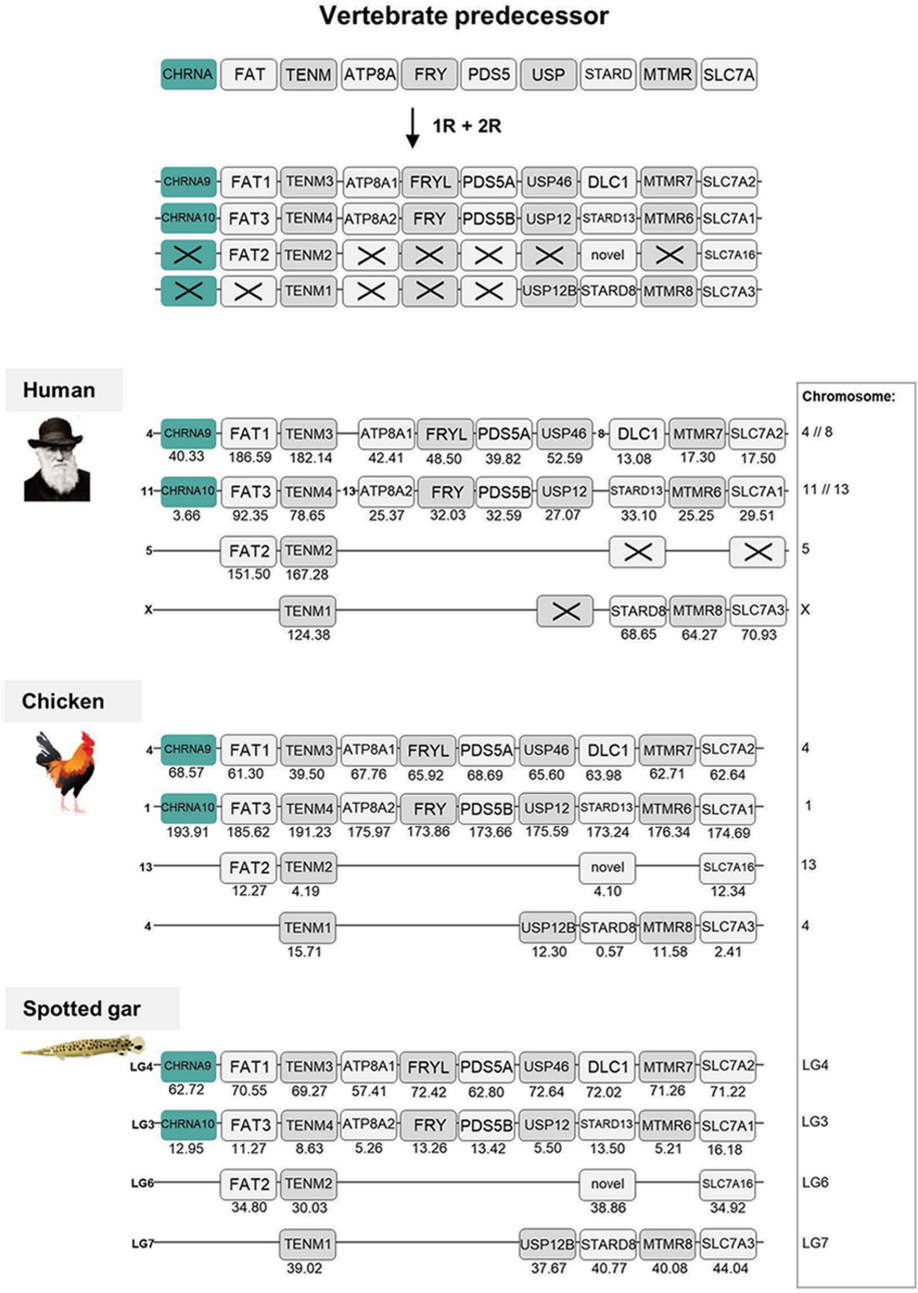
Analysis of the evolutionary history of the nAChR family with chromosomal locations of the nAChR genes and their neighboring gene families in human, chicken and spotted gar. Crosses indicate gene loss or gene not yet identified. Chicken and spotted gar illustrations are re-used with permission from Daniel Ocampo Daza, source: www.egosumdaniel.se and the human image is re-used with permission from https://commons.wikimedia.org. The duplication scheme and neighboring gene families for the *CHRNA9/CHRNA10* genes. The right panel summarizes chromosomes included. This paralogon is referred to as paralogon 3 in this study.

Next the *CHRNA9* and *CHRNA10* genome regions were analyzed. Following exclusion criteria stated in methods section, nine gene families were analyzed and included, namely *FAT, TENM, ATP8A, FRY, PDS5, USP, STARD, MTMR* and *SLC7A*. As in Fig. 3, following the phylogenetic analyses the chromosomal position of each gene family member was noted for human, chicken and spotted gar (Fig. 5). Three of the gene families in this region have retained all four copies in chicken and spotted gar (*TENM, STARD* and *SLC7A*) whereas in the human genome the *STARD* and *SLC7A* families are triplets. The other neighboring families have lost one or two members in all three species. As not so many gene families with three or four members were found in these genomic regions, also families with two members were included in the analysis. However, despite the smaller data set and some losses, this analysis supports gene family expansions through the 1R and 2R events. As in paralogon 1, there has been a translocation in human where the first member of the paralogon has gene families located on chromosome 4 or 8, and the second paralogon member is comprised by chromosomes 11 and 13, but in chicken and spotted gar each paralogon member is located on a single chromosome. According to the classification provided in [15] this set of chromosomes corresponds to paralogon C, here referred to as paralogon 3 (Fig. 5).

**Fig. 6:**
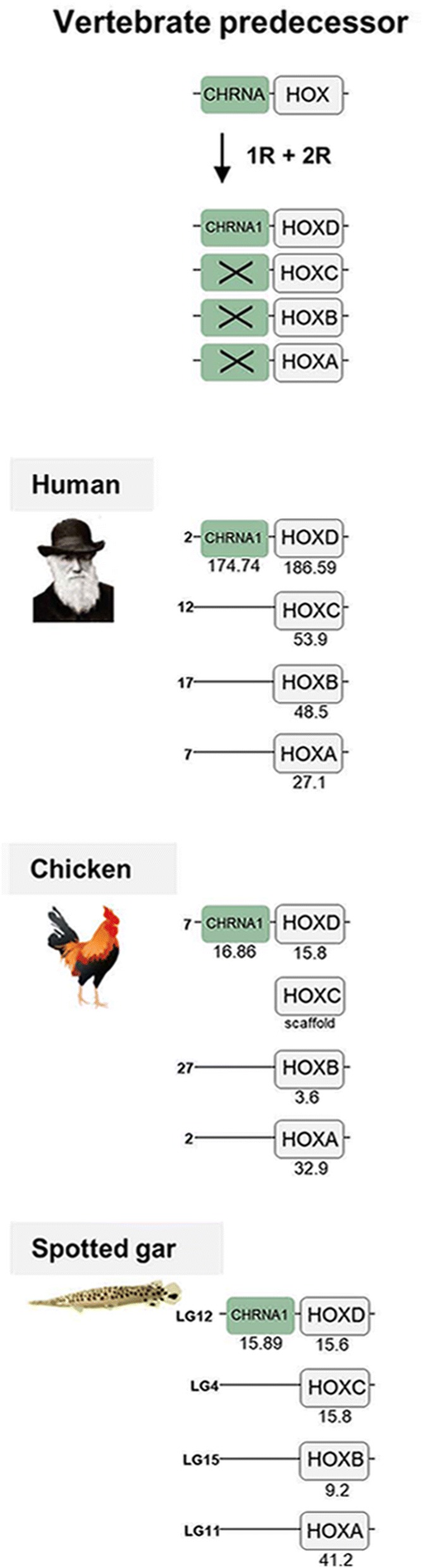
Analysis of the evolutionary history of the nAChR family with chromosomal locations of the nAChR genes and their neighboring gene families in human, chicken and spotted gar. Crosses indicate gene loss or gene not yet identified. Chicken and spotted gar illustrations are re-used with permission from Daniel Ocampo Daza, source: www.egosumdaniel.se and the human image is re-used with permission from https://commons.wikimedia.org. The duplication scheme and neighboring gene families for the *CHRNA1* gene. This region has been analyzed in detail in previous studies from our lab, see Larhammar et al. (2002), Sundström et al. (2008) and Widmark et al. (2011). This paralogon is referred to as paralogon 4 in this study

The *CHRNA1* gene is a single gene and has no 1R and 2R paralogs remaining today. It is located in close proximity to the *HOXD* cluster in human, chicken and spotted gar, belonging to paralogon E according to the classification in [15] and here referred to as paralogon 4 (Fig. 6). This paralogon has been analyzed in great detail previously [24–26].

**Fig. 7:**
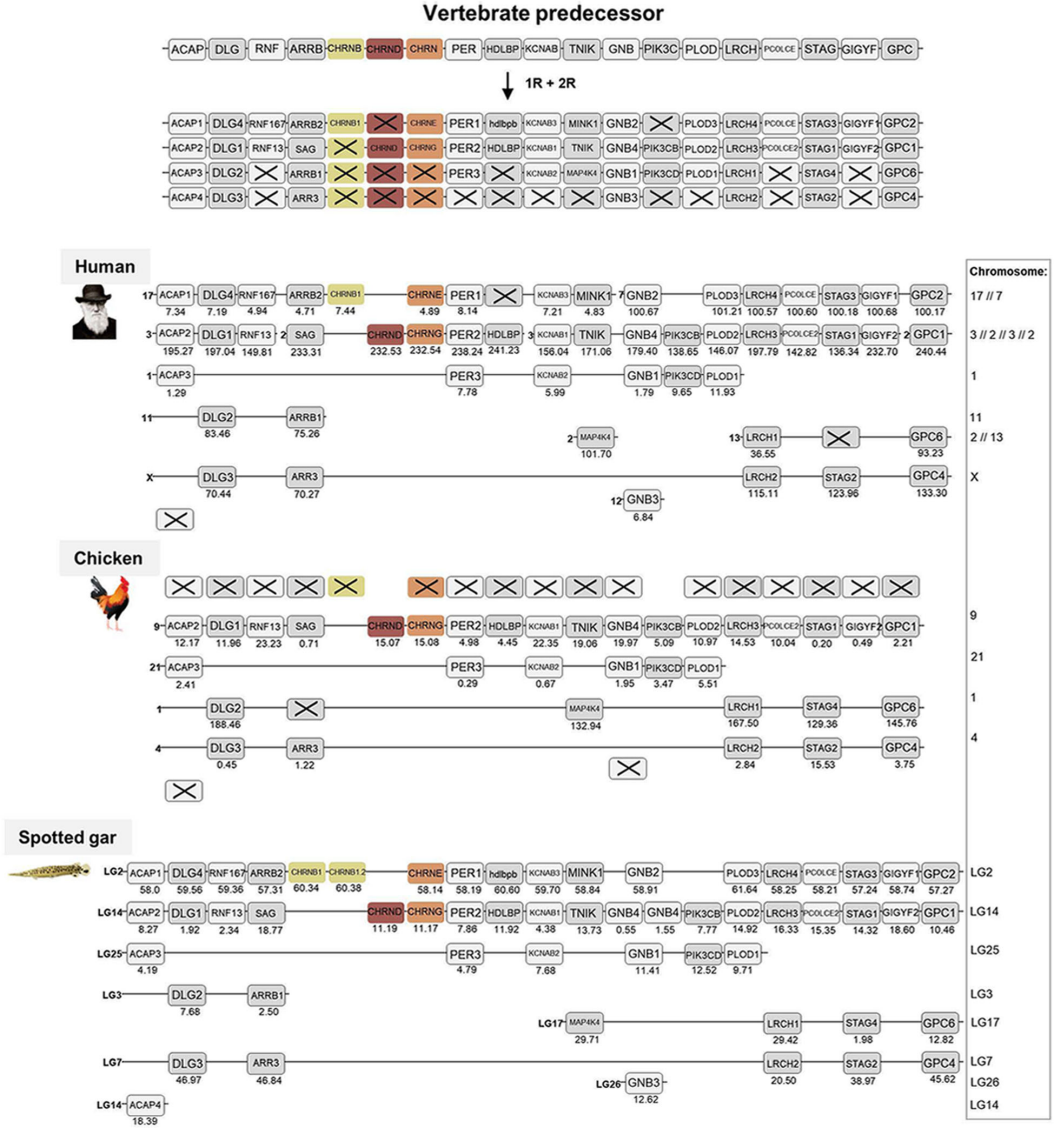
Analysis of the evolutionary history of the nAChR family with chromosomal locations of the nAChR genes and their neighboring gene families in human, chicken and spotted gar. Crosses indicate gene loss or gene not yet identified. Chicken and spotted gar illustrations are re-used with permission from Daniel Ocampo Daza, source: www.egosumdaniel.se and the human image is re-used with permission from https://commons.wikimedia.org. The neighboring gene families and duplication schemes for the *CHRNB1, CHRND* and *CHRNE/CHRNG* genes. The spotted gar retained a local duplication of the *CHRNB1* gene, forming *CHRNB1.2*. The right panel summarizes chromosomes included. This paralogon is referred to as paralogon 5 in this study

The remaining NMJ genes are located on human chromosomes 2 (*CHRNB1* and *CHRNE*) and 17 (*CHRND* and *CHRNG*). As already stated, duplicates seem not to have been retained for these genes after 1R and 2R, except for the duplication resulting in the *CHRNG* and *CHRNE* genes. Following analyses of the chromosomal regions surrounding these genes, 16 gene families were included namely: *ACAP, DLG, RNF, ARRB* (for phylogenetic analysis see previous study [27]), *PER, HDLBP, KCNAB, TNIK, GNB* (for phylogenetic analysis and chromosomal positions see previous study [28]), *PIK3C, PLOD, LRCH, PCOLCE, STAG, GIGYF* and *GPC*. After phylogenetic analyses, the chromosomal regions are displayed in Fig. 7. As this is a region that has been subject to many translocations, the analyses become more complicated and in order to gain as much information as possible also families with only two members were included in the analyses. These genomic regions seem to be a mixture of paralogons B and F, according to the classification in Nakatani et al. [15] which further indicates the complexity of the regions, here referred to as paralogon 5 (Fig. 7). However, despite these complications our analyses find nothing that would argue against gene family expansions through the 1R and 2R events. This figure also shows that spotted gar LG2 has a local gene duplication of the *CHRNB1* gene resulting in *CHRNB1.2* in the ancestor of ray-finned fishes as discussed above.

### The nAChR family expansion after the teleost specific tetraploidization

**Fig. 8:**
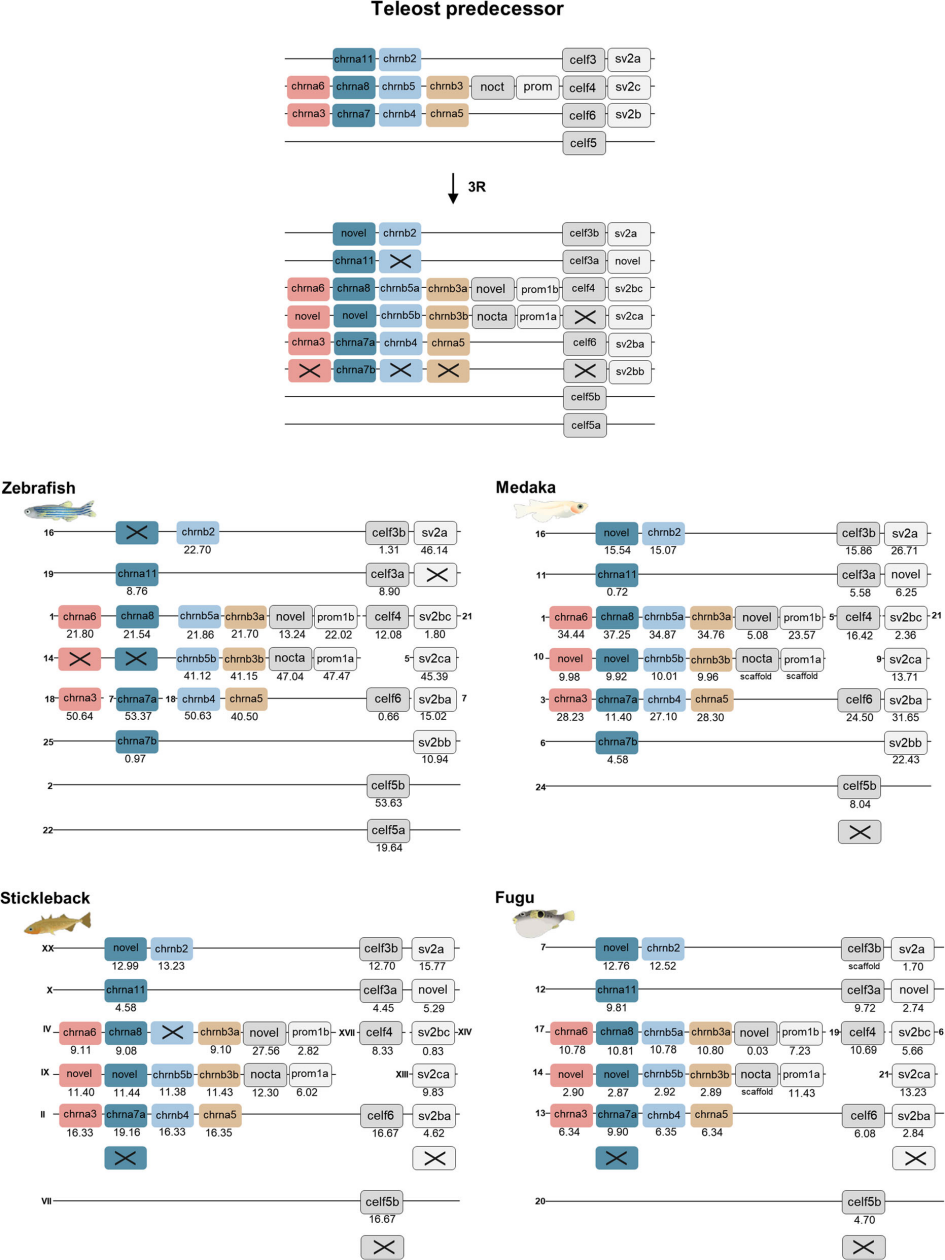
Analysis of the evolutionary history of the nAChR family with chromosomal location of the nAChR genes and their neighboring gene families in zebrafish, medaka, stickleback and fugu. Crosses indicate gene loss or gene not yet identified. Species illustrations are re-used with permission from Daniel Ocampo Daza, source: www.egosumdaniel.se

The phylogenetic analyses of the nAChR genes (Fig. 1, Additional file 1) indicate that in total 11 of the 19 nAChR genes in the vertebrate ancestor after 2R have retained duplicates in at least one of the teleosts (*CHRNA9*, *CHRNA10*, *CHRNA7*, *CHRNA8*, *CHRNA11*, *CHRNB5*, *CHRNA1*, *CHRNA2*, *CHRNA4*, *CHRNA6* and *CHRNB3*). From the phylogenetic tree these duplications could be interpreted as having occurred at the time of the teleost tetraploidization 3R. The obvious exception is *CHRNB1.2* which is a local duplicate. However, in order to verify that these duplicates are results of 3R a similar analysis as for the two initial vertebrate tetraploidizations was carried out for the paralogon with the largest number of nAChR genes, namely paralogon 1. Two neighboring gene families from the analysis of chromosomal regions in relation to 1R and 2R, with members present in zebrafish, medaka, stickleback and fugu, were chosen for an in depth analysis in teleosts; the *CELF* and *SV2* gene families (Fig. 8). To be noted here is that the *CELF4* and the *SV2C* duplicates are located on separate chromosomes in all teleost species included. This was also noted in the 1R and 2R synteny analysis, where these genes are located on separate chromosomes in human and spotted gar, whereas in chicken they are located in the same chromosomal region (Fig. 3) which shows that despite translocations the chromosomal regions belong to the same paralogon. In order to complement the analysis, the *NOCT* and *PROM* gene families were added. As this analysis shows that the duplicates analyzed are located in chromosomal regions harboring the same repertoire of gene families, we conclude that together with the phylogenetic results, the most parsimonious interpretation is that the duplicate nAChR genes in the teleosts are a result of 3R.

**Fig. 9:**
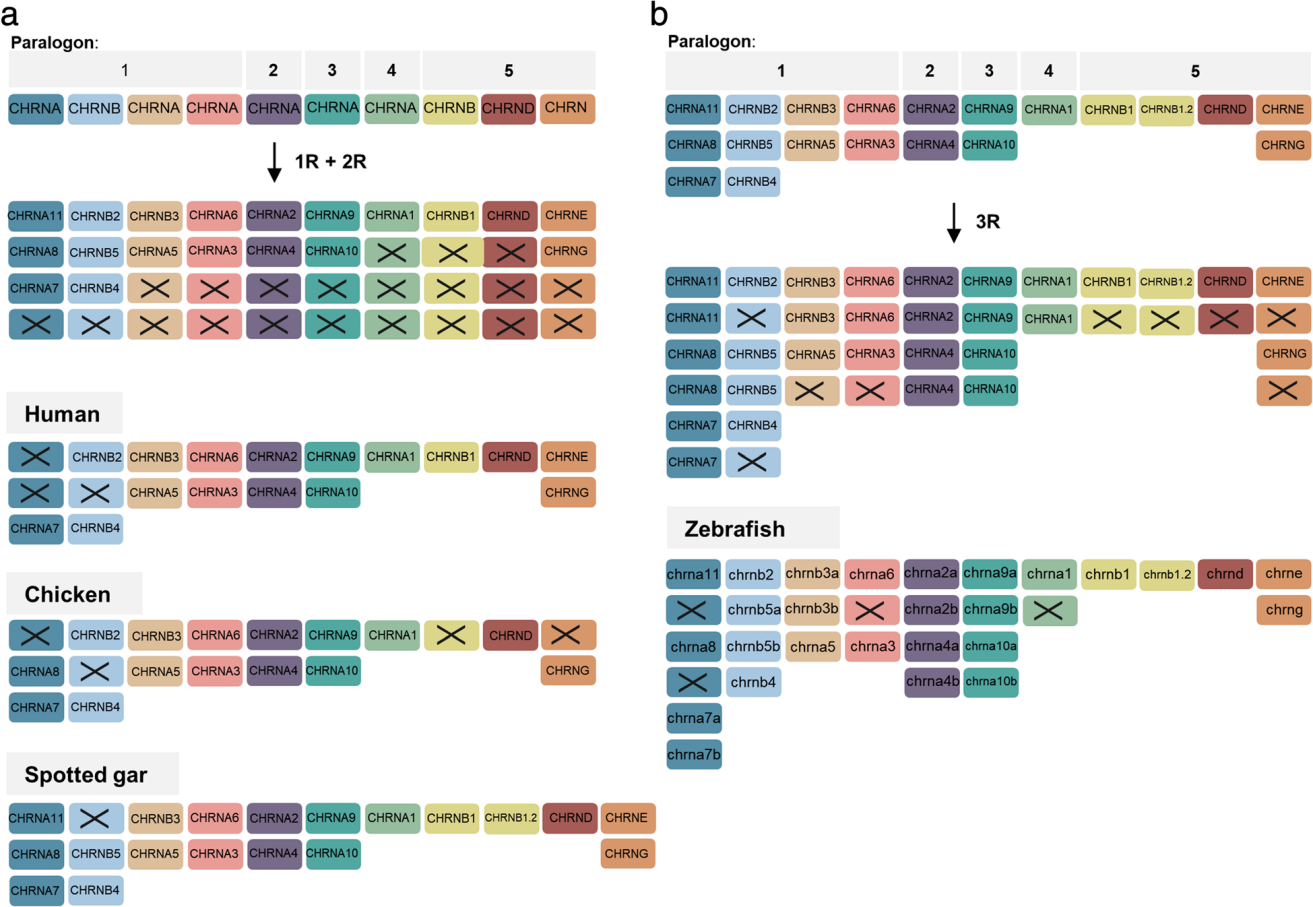
**a** Duplication scheme of the nAChR genes following 1R + 2R. Ten nAChR genes in five different paralogons are present in the vertebrate predecessor. Following 1R + 2R the repertoire expands to 19 genes. Human retained 16 genes (lacking *CHRNA11*, *CHRNA8* and *CHRNB5*), chicken 15 (lacking *CHRNA11*, *CHRNB5*, *CHRNB1* and *CHRNE*) and spotted gar 18 (lacking *CHRNB2*), plus the *CHRNB1.2* local duplication. **b** Duplication scheme of the nAChR genes following 3R. 20 nAChR genes are present in the teleost predecessor, following 3R the repertoire expands to 31 genes. Zebrafish has retained 27 genes. Crosses indicate gene loss or gene not yet identified

These results for the nAChR genes are summarized in Fig. 9. Our analyses show that the nAChR family consists of 10 subfamilies as defined by the ancestral repertoire of genes before 1R. The ancestral genes of these subfamilies were duplicated during the vertebrate tetraploidizations and following losses resulted in 19 ancestral vertebrate genes. Subsequently, mammals lost 3 additional genes resulting in 16 subunit genes present in humans today. Chicken seems to have lost two of these and two more, resulting in 15. The spotted gar has retained 18 of the ancestral 19 (lost only *CHRNB2*) and has gained one additional copy, the local *CHRNB1.2* duplicate (Fig. 8a). Following the teleost tetraploidizations, 20 genes (including the *CHRNB1.2* local duplicate) present in the teleost predecessor expanded to 31 nAChR genes present in the teleost ancestor (Fig. 8b). A few losses subsequently resulted in 27 nAChR genes present in zebrafish today, 28 in medaka, 27 in stickleback and 28 in fugu (Additional file 4).

## Discussion

The nAChR genes constitute a complex family structurally, evolutionarily as well as functionally [29–32]. As described in the introduction, the evolution of the nAChR genes has remained unresolved despite several efforts to deduce the order of gene duplications and to determine their time points. In addition to the approaches used previously when studying the evolution of this family, i.e. sequence-based phylogeny and exon-intron organization, we have also analyzed the chromosomal positions of the genes, i.e. conserved synteny and paralogous regions. This and the large number of vertebrate genomes from many taxa now available facilitates more detailed analysis than previously possible.

Using this approach, we are able to pinpoint accurately when many of the duplications took place, namely as a result of the two basal tetraploidizations at the origin of the vertebrates. This conclusion results from our analysis of the repertoire of neighboring genes on the chromosomes of the different nAChR genes. The basis for this approach is that if several gene families have members in multiple chromosome regions, the most parsimonious explanation is that all these families were duplicated simultaneously by a chromosome (or genome) doubling event. This has been convincingly shown to be the case for a large proportion of the human genome by comparison of chromosomal regions within the human genome and with many other vertebrate species [15–17].

Our analyses of protein sequences and exon-intron organization are shown in Fig. 1 and Fig. 2 and a summary of our paralogon analyses is presented in Fig. 8. Taken together, these three data sets show that the number of nAChR genes present before the two basal vertebrate tetraploidizations 1R and 2R, i.e., the nAChR repertoire of the vertebrate predecessor, was 10. If the three NMJ non-α subunits are considered a single clade, the tree in Fig. 1 shows that all eight clades are anchored by basally diverging invertebrate deuterostome sequences (amphioxus, tunicates), supporting the concept of 10 ancestral genes giving rise to 10 subfamilies (the NMJ subunit genes will be discussed further below).

The 10 genes in the vertebrate predecessor increased to 19 via the 1R and 2R tetraploidizations. The combined results, and especially those from our synteny and paralogon analyses, show that the nine additional genes that have arisen in vertebrates with 1R and 2R can be explained by the basal tetraploidizations and thereby can be fixed in time to these events. Additional duplications that occurred as a result of the teleost 3R event will be discussed below. Only two presently known vertebrate nAChR genes did not arise by tetraploidization, namely ray-finned fish *CHRNB1.2* as a local duplicate of *CHRNB1*, and the frog *Xenopus tropicalis* *CHRNA1* duplicate.

The double tetraploidization scenario means that there may have been as many as 21 losses of duplicates, or fewer if duplicates were lost already after 1R. None of the ancestral genes have kept all four ohnologs, i.e. all four copies resulting from 1R and 2R. Two of them have retained three ohnologs (i.e., tetraploidizations duplicates), the *CHRNA7/CHRNA8/CHRNA11* and the *CHRNB2/CHRNB4/CHRNB5* clades, whereas five of the ancestral genes have retained two ohnologs (Fig. 8a). Three ancestral genes have remained single genes following 1R and 2R, i.e., none of their ohnologs have been retained. Regarding the two triplicates, the ancestral gene must have duplicated in 1R and then both copies were duplicated in 2R after which one was lost. Regarding the ancestral nAChR genes that are present as ohnolog pairs, for instance *CHRNA2/CHRNA4* (Fig. 8a), there are two possible scenarios. One scenario is that the ancestral gene was duplicated in 1R resulting in two genes. These two genes were then duplicated in 2R, resulting in four genes, after which two were lost. The other scenario is that the ancestral gene was duplicated in 1R, resulting in two genes, after which one was lost. Then 2R duplicated the remaining gene, resulting in the two ohnologs present today. As the two tetraploidizations were probably quite close in time, we cannot say if an ohnolog pair is the result of one loss after 1R or two losses after 2R.

The 10 ancestral nAChR genes are distributed among no less than five paralogons. Assuming that the 10 pre-vertebrate genes were generated by local gene duplications from a common ancestral gene, which is a frequent type of duplication, sufficient time has apparently passed for several of the duplicates to be translocated to separate chromosomes before the basal vertebrate tetraploidizations. The most recent duplications prior to 1R, i.e. the duplications that generated the ancestors of *CHRNA3/CHRNA6, CHRNB2/CHRNB4/CHRNB5* and *CHRNA5/CHRNB3* on the one hand and the ancestors of *CHRNB1, CHRND* and *CHRNE/CHRNG* on the other, are located close together on their respective chromosome, most likely as a result of local gene duplications. Perhaps their close proximity to each other even indicates some degree of co-regulation of gene expression.

In the analyses of the evolution of the nAChR family about 20 years ago, three main vertebrate subsets were proposed: the NMJ genes *CHRNB1/CHRND/CHRNG/CHRNE*, the neuronal *CHRNA2-CHRNA6/CHRNB2-CHRNB4* genes and the *CHRNA7/CHRNA8* genes [9, 10]. Subsequently a fourth subset containing the *CHRNA9/CHRNA10* genes was added [11, 12]. The relationship of the *CHRNA1* gene has been contentious. Initially it was grouped together with the neuronal subunit genes [9–11]. A later analysis instead grouped *CHRNA1* closest to the other NMJ genes [12]. Based on our phylogenetic analysis with a much broader representation of vertebrate species than in previous studies, the *CHRNA1* gene is grouped together with the *CHRNA2*-*CHRNA6*/*CHRNB3* genes, with maximal node support (Fig. 1). When analyzing the exon-intron organization, the *CHRNA1* gene has some similarity to the other NMJ genes resulting in two possible evolutionary scenarios for the *CHRNA1* gene. The first scenario is the same as the one resulting from the phylogenetic analysis, where the *CHRNA1* clusters with the neuronal α-genes, as shown in Figs 1 and 2. In this scenario, the introns 12 and 13 were inserted as separate events in *CHRNA1* and the other NMJ genes, respectively. The second scenario would instead cluster the *CHRNA1* gene together with the NMJ genes and its closest common ancestral gene would be shared with *CHRNB1*/*CHRNB1.2*. In this scenario, intron 13 was gained in the common ancestor of the NMJ family. Subsequently the common ancestor of *CHRNB1*/*CHRNB1.2* and *CHRNA1* received intron 12. The second scenario is the most parsimonious explanation based upon the exon-intron organization analyses, as it requires one event less. However, we find the first scenario to be more likely as it is based on extensive sequence-based phylogenetic analyses. If we would force the second scenario onto the sequence-based tree, it would mean that the *CHRNA1* sequence would need to move three nodes in order to cluster with the NMJ genes instead of the neuronal α-genes. These nodes all have maximum support in the ML tree, therefore it seems unlikely that they would not be true. We do not consider the positions of the inserted introns to argue strongly enough against the sequence analysis. Furthermore, as mentioned in Results, the *CHRNA1* has the cysteine pair encoded shortly before TM1 which is characteristic for the α-subunits but is absent in the non-α NMJ subunits. The chromosomal location of the *CHRNA1* gene gives no further clues to its relationships as it is located in a paralogon with no other nAChR genes (Fig. 8). In conclusion, we find that *CHRNA1* most likely arose as a member of the clade consisting of α-subunit genes rather than the clade containing the other NMJ genes.

Another interesting matter is the relationships of the other NMJ (non-α) subunit genes with one another, and foremost the *CHRND* gene which seems to have diverged before the appearance of the *CHRNE/CHRNG* genes. Although it is difficult to estimate an exact time point for this event, we believe that the *CHRND* gene was formed by a local duplication and hence diverged before 1R and 2R. The two tetraploidizations then generated the *CHRNE* and *CHRNG* genes from their common ancestral gene, whereas the *CHRND* duplicates were lost.

Surprisingly the *CHRNB1* and *CHRNE* genes are missing in the chicken genome assembly. However, as these subunits are necessary for functional receptors at the NMJs at least in mammals, it remains possible that the *CHRNB1* and *CHRNE* genes may be present on microchromosomes in chicken, i.e. parts of the genome which are difficult to sequence due to high GC-content [33, 34]. Figure 7 highlights the generality of this problem in the chicken, as all members expected to be located on one and the same chromosome are missing in the chicken genome assembly. Thus, either this whole chromosomal region has been lost in chicken or, alternatively, all or many of these genes have ended up on a microchromosome that is still unsequenced. This is a possibility that could apply to all nAChR genes that have not identified in certain species in this study - we cannot know whether this is due to loss of this gene in the species or if it is due to low sequence coverage in some parts of the genome such as GC-rich regions. Finally regarding the NMJ subunit genes, it would be interesting to further investigate the local duplicate of the *CHRNA1* gene in the frog, in order to see whether these two *CHRNA1* gene copies differ from each other in terms of anatomical expression pattern or developmental timing [19].

The duplication scenario proposed by us in 1998 was the first that took into consideration the two early vertebrate tetraploidizations [14]. It was based on the chromosomal positions of the 13 nAChR genes in the human genome that were known at the time, before the sequencing of the human genome and the genes had been mapped to chromosomal regions by others using experimental methods. We can now see that several more gene duplications preceded the two tetraploidizations than we realized at the time, and that more losses have occurred after 2R, as has been observed for several other gene families. Nevertheless, three of the four duplications that we proposed were due to 2R are indeed a result of the basal tetraploidizations.

Now that we can connect so many of the nAChR gene duplications to the 1R and 2R events, which are considered to have taken place close to one another probably in the time range between 550 and 500 million years ago, it is interesting to compare with duplication time points deduced from the degree of divergence of these gene pairs or triplets. In the report by [10], the duplications leading to the pairs *CHRNA2-CHRNA4, CHRNA3-CHRNA6, CHRNA5-CHRNB3, CHRNB2-CHRNB4* and *CHRNA7-CHRNA8* were all calculated to have taken place less than 300 million years ago. In the evolutionary tree presented by Le Novère et al. [12], these five duplications were estimated to have occurred between 430 and 850 million years ago. This shows the usefulness of the chromosome duplication information when rates of sequence divergence vary.

Following the teleost-specific tetraploidization of the 19 + 1 basal ray-finned fish nAChR genes, zebrafish has retained 7 duplicates, resulting in a total of 27 genes. The genes that have retained 3R ohnologs are *CHRNA2, CHRNA4, CHRNA7, CHRNA9, CHRNA10, CHRNB5* and *CHRNB3*. In the other teleost species that we have investigated, the situation is slightly different: medaka has in total 28 genes, stickleback has 27 and fugu has 28 genes (Additional file 4). The 28 genes in fugu correspond to the number of genes identified in a previous study [35], however with genomic information from the newest fugu genome assembly, we can now with higher precision say which gene is which. Interestingly, the only genes for which 3R ohnologs are kept in all teleost species investigated are *CHRNA9*, *CHRNA10* and *CHRNB3.* Among the other 3R ohnologs, there are species differences in the repertoire. From this analysis it can be concluded that the teleost ancestor present before the teleost radiation had a total of 31 nAChR genes, thus 11 3R ohnologs. Particularly interesting to investigate further among these would be *CHRNA4* and *CHRNA7*. The *CHRNA4* gene encodes one of the most abundantly expressed nAChR subunits in the brain. The α7 subunit can function as a homopentamer and has recently been reported to also form heteropentamers in the brain [7]. As these subunit genes with extra 3R paralogs are expressed in the hippocampus, it will be interesting to investigate their functional roles in for instance learning and memory.

Finally, we also discovered a previously undescribed gene, the *CHRNA11* which is also present in all three slowly evolving genomes included in our species panel, namely spotted gar, coelacanth and Australian ghostshark. It is also present in teleosts: zebrafish has one copy whereas a 3R duplicate has been retained in medaka, stickleback and fugu. Paralogon analysis showed that *CHRNA11* arose in the vertebrate 1R and 2R tetraploidizations and shares a common ancestor with *CHRNA7* and *CHRNA8*. As zebrafish is a common and important model organism in research today, it is crucial to know its full repertoire of nAChR genes when investigating its cholinergic system.

## Conclusions

In conclusion, our analyses using three separate approaches, i.e., sequence phylogeny, exon-intron organization and chromosomal information for synteny and paralogons, have allowed us to arrive at a gene duplication scenario where tetraploidizations account for all duplications of nAChR genes in vertebrates except two (one local duplicate in the ray-finned fish ancestor and one in the frog *Xenopus tropicalis*). The predecessor of the vertebrates increased from 10 ancestral genes to 19 after 1R and 2R. In the teleost ancestor, the 19 + 1 members became 31 via the 3R tetraploidization. Thus, it would seem logical to consider the 10 ancestral pre-vertebrate genes as the founders of 10 subfamilies, of which seven gained additional members in 2R. Now that the time points of the gene duplications have been determined, it will be interesting to see how the expression patterns and the temporal regulation of the members within each subfamily may have sub- or neo-functionalized.

## Methods

### Species sequences

Species sequences included in the nAChR family analyses were human (*Homo sapiens*; Hsa), mouse (*Mus musculus*; Mmu), opossum (*Monodelphis domestica;* Mdo), chicken (*Gallus gallus*; Gga), anole lizard (*Anolis carolinensis*; Aca), frog (*Xenopus tropicalis*; Xtr), coelacanth (*Latimeria chalumnae*; Lch), spotted gar (*Lepisosteus oculatus*; Loc), zebrafish (*Danio rerio*; Dre), stickleback (*Gasterosteus aculeatus*; Gac), medaka (*Oryzias latipes*; Ola), fugu (*Takifugu rubripes;* Tru) and Australian ghostshark (*Callorhinchus milii*; Cmi). For the *CHRNE* and *CHRNA4* genes another frog species was used (*Nanorana parkeri;* Npa). For the *CHRNA3, CHRNA5* and *CHRNB4* genes Chinese softshell turtle (*Pelodiscus sinensis*; Psi) was included. For the *CHRNB1* and *CHRNE* genes, additional sequences were included from Chinese softshell turtle, Burmese python (*Python bivittatus*; Pbi) and American alligator (*Alligator mississippiensis;* Ami). For outgroups to the nAChR subfamilies, sequences from amphioxus (*Branchiostoma floridae*; Bfl), tunicates (*Ciona intestinalis*; Cin and *Ciona savignyi*; Csa), nematode (*C. elegans;* Cel) and fruitfly (*Drosophila melanogaster;* Dme) were included.

### Retrieval of amino acid sequences

For phylogenetic analyses, the amino acid sequences were retrieved from the Ensembl genome browser (release 84) [36, 37] and NCBI [38] databases. If sequences were not found in either of the databases, the sequence of the most closely related species (out of the pre-selected species for this study), was used as query sequence in a TBLASTN search and the sequences that were found in the search were included in the analysis if found to be unique orthologs. For outgroup sequences from amphioxus, *Ciona intestinalis* and *ciona savignyi* TBLASTN results were controlled against human sequence of the 5-hydroxytryptamine receptor 3A (HTR3A) and 3B (HTR3B) and if grouped into the same clade in the neighbor joining (NJ) tree, the sequences were categorized as non-nAChR and excluded from further analysis. The NJ tree was generated with standard settings, the random number generator seed was set to 500 and the number of bootstrap trials was set to 1000. As outgroup for the complete nAChR family, the human 5HTR3A and 5HTR3B sequences were included. Information about sequences included in the analysis is found in Additional file 2.

### Multiple sequence alignments and phylogenetic analyses

Jalview 2.10.1 was used with Muscle default settings for sequence alignment [39]. If the amino acid sequences were aligning poorly and the predictions appeared questionable, the genomic sequences were investigated and the sequences were manually edited and annotated, by comparing sequence homology and consensus donor and acceptor splice sites. In regions of high variability, such as the intracellular domain of the amino acid sequence, the sequence alignment appeared shifted in a few instances and these were adjusted manually. Such manual adjustments were kept to a minimum. The CLUSTAL [39] and PRANK (available at the EMBL-EBI website [40]) alignment algorithms were also tested as controls [39]. Then, a phylogenetic maximum likelihood analysis was performed using the IQ TREE 1.6.3 application [41, 42]. The ModelFinder was used for selection of the optimal substitution model [43], resulting in JTT + I + G4 according to BIC. Node supports were calculated using the non-parametric UltraFast Bootstrap (UFBoot) method [44] and the SH-aLRT branch test with 1000 replicates. The resulting tree was displayed in FigTree v1.4.2 and rooted with the human 5HTR3A and 5HTR3B genes. The raw data phylogenetic tree is available in nexus format in the Dryad Digital repository [21].

### Exon-intron organization

For protein domain identification and determination of domain boundaries, the Pfam 31.0 web page was used ([45], http://pfam.xfam.org/). The exon-intron boundaries marked in Fig. 2 are based on the human genes. For the genes absent in humans (i.e. *CHRNB1.2, CHRNB5, CHRNA8* and *CHRNA11*) the spotted gar sequences were used as templates. In the spotted gar *CHRNA11* gene, exon 3–6 was missing and therefore the zebrafish *CHRNA11* gene is shown in Fig. 2. Sequences with a common exon-intron organization were grouped together. Positions of structurally important features such as cysteines, cysteine-pairs and N-linked glycosylation sites were identified in the Jalview alignment and for each receptor subtype they were compared across all vertebrate orthologs included in the analysis.

### Conserved Synteny and paralogon analysis in relation to 1R and 2R

For synteny and paralogon analyses, the neighboring regions of the nAChR genes were investigated in human, chicken and spotted gar. Gene lists of the genomic regions 10 Mb upstream and downstream of the CHRNA11/CHRNB2, CHRNA6/CHRNA8/CHRNB5/CHRNB3, CHRNA3/CHRNA7/CHRNB4/CHRNA5, CHRNA9, CHRNA10, CHRNB1/CHRNE and CHRND/CHRNG genes in spotted gar were downloaded using the Biomart function in Ensembl version 83. From the gene lists gene families with at least two members were selected for synteny analysis. Gene families with an unclear topology and/or weak node supports in the phylogenetic analyses, and families with a high degree of sequence conservation and/or lack of outgroups, were not included in the analysis. Vertebrate species included in the phylogenetic analyses were human, chicken, coelacanth, spotted gar and zebrafish. As outgroups cionas, amphioxus, fruitfly, *C. elegans* and in some cases other human gene sequences were used. aLRT SH-like trees were constructed using the PhyML 3.0 web server [46, 47] in order to verify the sequence orthology. To apply the most optimal substitution model the “Automatic model selection by SMS” was selected, with Akaike Information Criterion. NNI was used as tree improvement method. The neighboring gene family information and aLRT trees are available in the Dryad Digital repository [21]. The regions including *CHRNA2, CHRNA4* and *CHRNA1* have been analyzed in depth previously in our lab [22–26]. The resulting gene families in human, chicken and spotted are presented in synteny figures (Figs. 3 to 7) showing the evolutionary schemes of the nAChR genes and neighboring gene families.

### Conserved Synteny and paralogon analysis in relation to 3R

For analysis of the chromosomal positions of the CHRNA11/CHRNB2, CHRNA6/CHRNA8/CHRNB5/CHRNB3 and CHRNA3/CHRNA7/CHRNB4/CHRNA5 genes in relation to 3R the CELF and SV2 gene families included in the analysis in relation to 1R and 2R were selected and analyzed in zebrafish, medaka, stickleback and fugu. In addition new gene lists were created according to the description above except in zebrafish. aLRT SH-like trees were constructed as described above. The first two gene families with correct phylogeny according to the Ensembl phylogenetic trees as well as genes present in all fish species analyzed were selected and included in synteny figure (Fig. 8). The neighboring gene family information and aLRT trees are available in the Dryad Digital repository [21].

## Additional files

Additional file 1: Phylogenetic maximum likelihood tree of the nAChR genes, rooted with the human 5HTR3A and 5HTR3B (root not shown). Complete tree is shown followed by zooms of all subfamilies. The tree topology is supported by a non-parametric Ultra-Fast Bootstrap (UFBoot) and approximate Likelihood-Ratio Test (aLRT) with 1000 replicates. For simplicity only UFBoot values are shown in the figure. The taxa ID contains the species, the localization of the gene (chromosome/scaffold/contig number) and a number indicating the order on the chromosome/scaffold/contig if several genes are located on the same one. The taxa ID of human and zebrafish genes with annotated HGNC or ZFIN names, respectively, also contain these names. The assigned taxa ID and sequence information details are provided in Additional file 2. (PDF 1490 kb)

Additional file 2: Table containing information about the nAChR gene sequences included in the analysis. First, information about genome assembly versions used are listed, then the following information about the nAChR genes is provided: species, common or assigned name, HGNC/ZFIN/Flybase symbol name, chromosome or genomic scaffold position, orientation, Ensembl ID or NCBI accession number, transcript count, assigned taxa ID and additional comments regarding sequence update date on NCBI or if there has been manual edits of the original Ensembl or NCBI sequence. The order of sequences is organized according to orthology. (XLSX 40 kb)

Additional file 3: Multiple sequence alignment file of the nAChR amino acid sequences included in analysis. The taxa ID is standardized as in Additional file 1. (DOC 395 kb)

Additional file 4: The nAChR gene repertoire in the four teleost species zebrafish, medaka, stickleback and fugu. The zebrafish has retained 27 genes, the medaka and stickleback 28 and the fugu 29 nAChR genes. The teleost ancestor had 31 nAChR genes. (PDF 192 kb)

## Abbreviations

5-HT: 5-hydroxytryptamine
ACh: Acetylcholine
aLRT SH: Approximate likelihood ratio test Shimodaira–Hasegawa
BD: Binding domain
CHRN: Cholinergic receptor nicotinic
ECD: Extracellular domain
ICD: Intracellular domain
nAChR: Nicotinic acetylcholine receptor
NJ: Neighbor joining
NMJ: Neuromuscular junction
OMIM: Online mendelian inheritance of man
TM: Transmembrane
UFBoot: UltraFast Bootstrap
WGD: Whole genome duplication
