# Supplementary material for: Evolution of vertebrate nicotinic acetylcholine receptors
Source: BMC Evol Biol. 2019 Jan 30;19:38. doi: 10.1186/s12862-018-1341-8 (PMC6354393; doi:10.1186/s12862-018-1341-8)
Supplement: Supplementary file 1 — Phylogenetic maximum likelihood tree of the nAChR genes, rooted with the human 5HTR3A and 5HTR3B (root not shown). Complete tree is shown followed by zooms of all subfamilies. The tree topology is supported by a non-parametric Ultra-Fast Bootstrap (UFBoot) and approximate Likelihood-Ratio Test (aLRT) with 1000 replicates. For simplicity only UFBoot values are shown in the figure. The taxa ID contains the species, the localization of the gene (chromosome/scaffold/contig number) and a number indicating the order on the chromosome/scaffold/contig if several genes are located on the same one. The taxa ID of human and zebrafish genes with annotated HGNC or ZFIN names, respectively, also contain these names. The assigned taxa ID and sequence information details are provided in Additional file 2. (PDF 1490 kb) [file 12862_2018_1341_MOESM1_ESM.pdf]

**Introduction to Additional file 1:**

The tree topology for each receptor subtype generally agrees quite well with the established species phylogeny. For a few of the subtypes the position of the chicken and/or lizard branches deviate from the species phylogeny, e.g. *CHRNA10, CHRNA11, CHRNG, CHRNB2, CHRNA1, CHRNB3, CHRNA5, CHRNA3, CHRNA6* and *CHRNA2* probably due to few species in these vertebrate classes. For *CHRN8* the frog sequence should form a clade with chicken and lizard. For *CHRNA11* the Australian ghostshark should be the first sequence to branch off. For *CHRNB1* and *CHRNE* the reptile sequences do not form one clade, probably because they differ considerably in their evolutionary rates. For *CHRNE, CHRNB2, CHRNA1, CHRNA5* and *CHRNA2* the coelacanth sequence should cluster with the tetrapods and for *CHRNA3* coelacanth should branch off before the frog sequence. For the *CHRNB4/CHRNB2/CHRNB5* clade, one Australian ghostshark sequence branches off first, although this node has low support and in previous ML tree version it has clustered together with *CHRNB4*. For *CHRNA4*, the opossum has an odd placement in the tree and the spotted gar sequence should cluster with the ray-fined fishes. Also, the zebrafish chrna4a gene differs considerably from its *chrn4b* paralog.

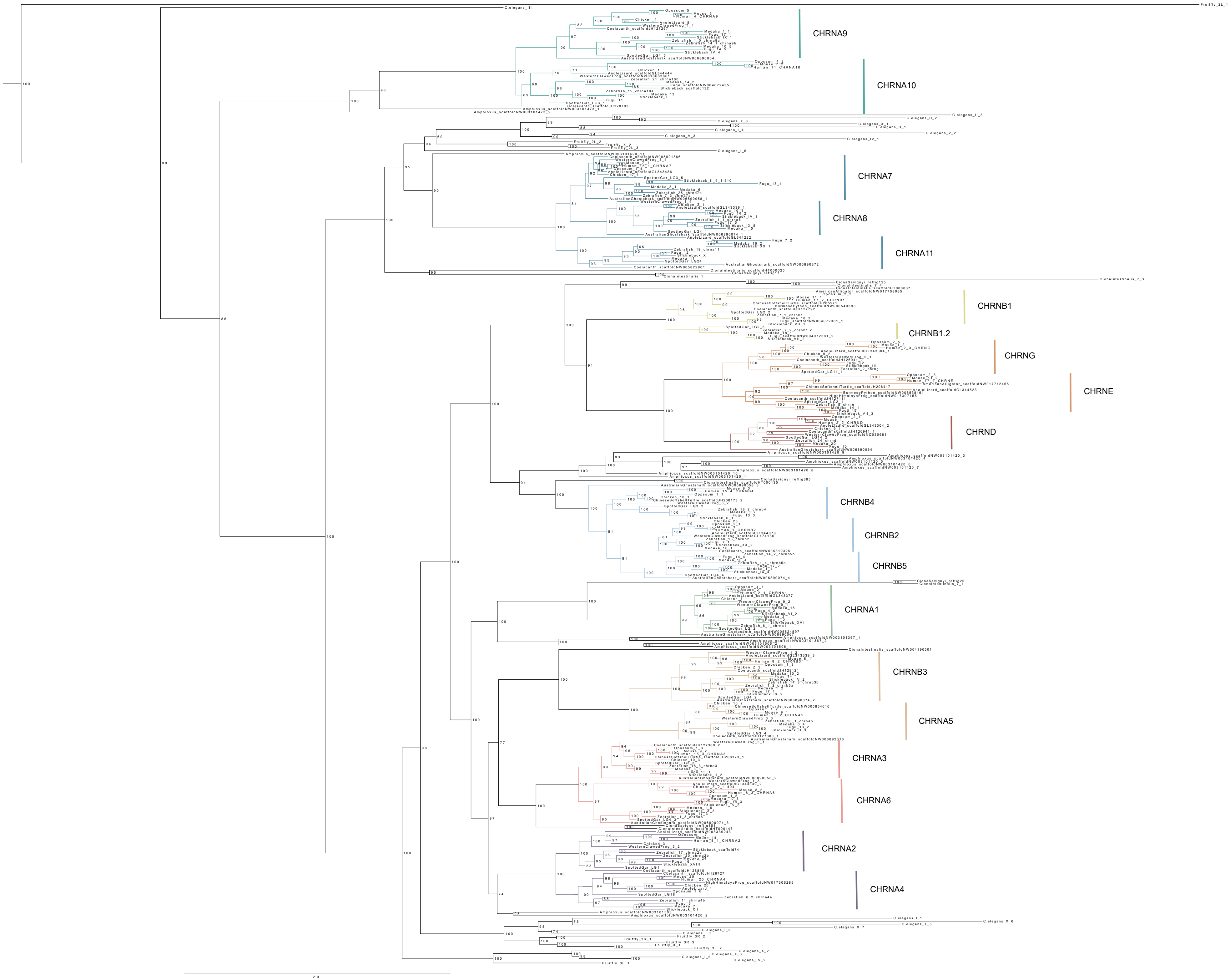
CHRNA9
CHRNA10
CHRNA7
CHRNA8
CHRNA11
CHRNB1
CHRNB1.2
CHRNG
CHRNE
CHRND
CHRNB4
CHRNB2
CHRNB5
CHRNA1
CHRNB3
CHRNA5
CHRNA3
CHRNA6
CHRNA2
CHRNA4
2.0

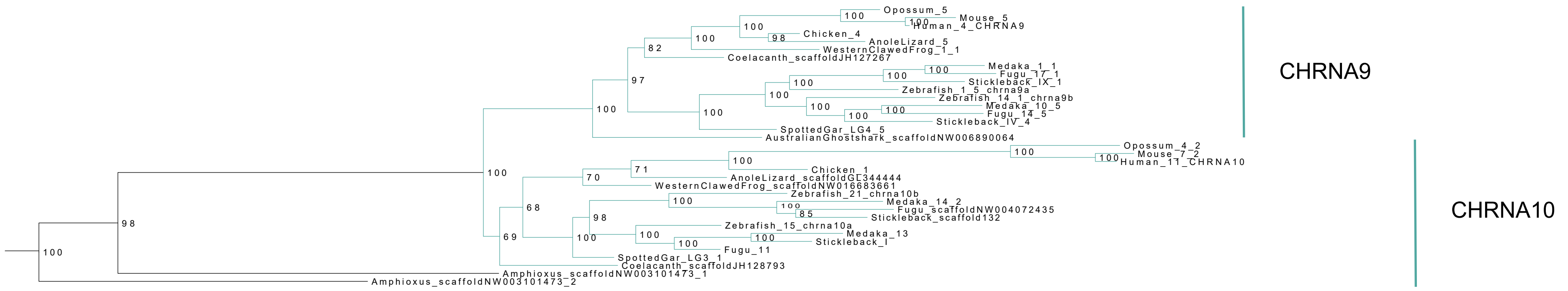
Opossum_5
Mouse_5
Human_4_CHRNA9
Chicken_4
AnoleLizard_5
WesternClawedFrog_1_1
Coelacanth_scaffoldJH127267
Medaka_1_1
Fugu_17_1
Stickleback_IX_1
Zebrafish_1_5_chrna9a
Zebrafish_14_1_chrna9b
Medaka_10_5
Fugu_14_5
Stickleback_IV_4
SpottedGar_LG4_5
AustralianGhostshark_scaffoldNW006890064
Opossum_4_2
Mouse_7_2
Human_11_CHRNA10
Chicken_1
AnoleLizard_scaffoldGL344444
WesternClawedFrog_scaffoldNW016683661
Zebrafish_21_chrna10b
Medaka_14_2
Fugu_scaffoldNW004072435
Stickleback_scaffold132
Zebrafish_15_chrna10a
Medaka_13
Stickleback_I
Fugu_11
SpottedGar_LG3_1
Coelacanth_scaffoldJH128793
Amphioxus_scaffoldNW003101473_1
Amphioxus_scaffoldNW003101473_2
CHRNA9
CHRNA10

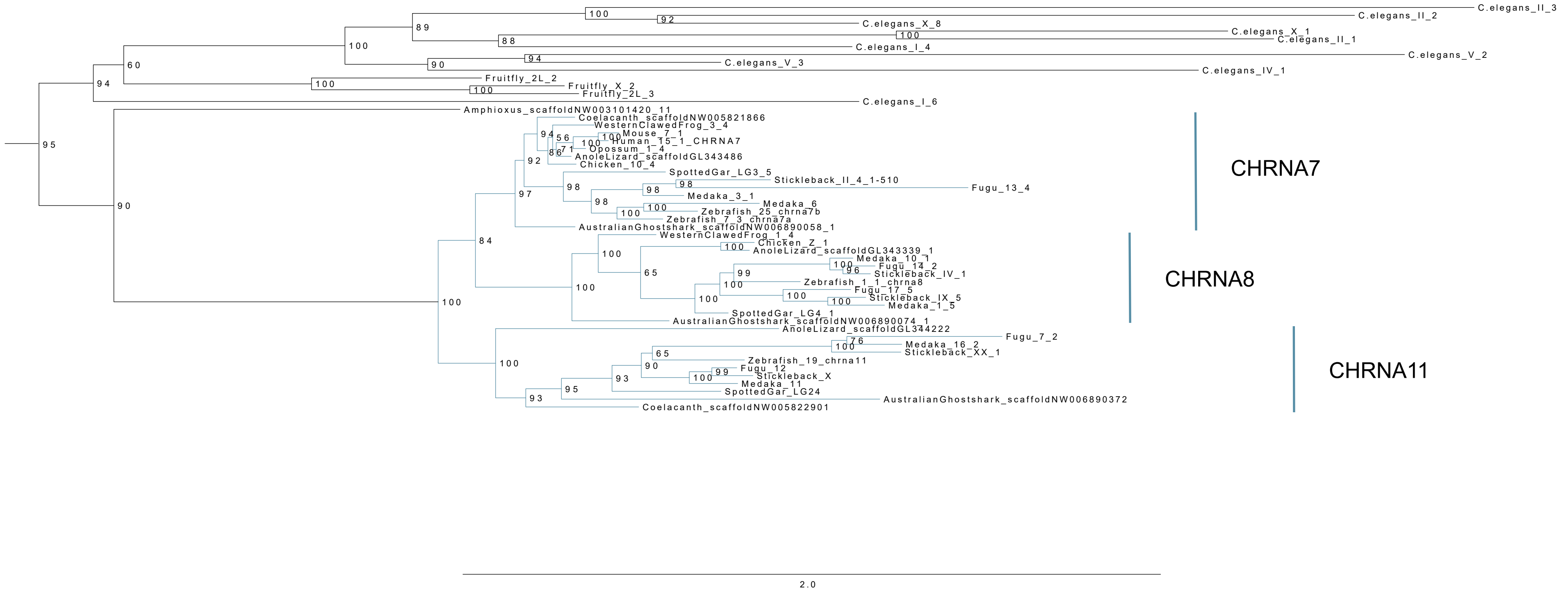
C.elegans_II_3
C.elegans_II_2
C.elegans_X_8
C.elegans_X_1
C.elegans_II_1
C.elegans_I_4
C.elegans_V_2
C.elegans_V_3
C.elegans_IV_1
Fruitfly_2L_2
Fruitfly_X_2
Fruitfly_2L_3
C.elegans_I_6
Amphioxus_scaffoldNW003101420_11
Coelacanth_scaffoldNW005821866
WesternClawedFrog_3_4
Mouse_7_1
Human_15_1_CHRNA7
Opossum_1_4
AnoleLizard_scaffoldGL343486
Chicken_10_4
SpottedGar_LG3_5
Stickleback_II_4_1-510
Fugu_13_4
Medaka_3_1
Medaka_6
Zebrafish_25_chrna7b
Zebrafish_7_3_chrna7a
AustralianGhostshark_scaffoldNW006890058_1
WesternClawedFrog_1_4
Chicken_Z_1
AnoleLizard_scaffoldGL343339_1
Medaka_10_1
Fugu_14_2
Stickleback_IV_1
Zebrafish_1_1_chrna8
Fugu_17_5
Stickleback_IX_5
Medaka_1_5
SpottedGar_LG4_1
AustralianGhostshark_scaffoldNW006890074_1
AnoleLizard_scaffoldGL344222
Fugu_7_2
Medaka_16_2
Stickleback_XX_1
Zebrafish_19_chrna11
Fugu_12
Stickleback_X
Medaka_11
SpottedGar_LG24
AustralianGhostshark_scaffoldNW006890372
Coelacanth_scaffoldNW005822901
CHRNA7
CHRNA8
CHRNA11
2.0

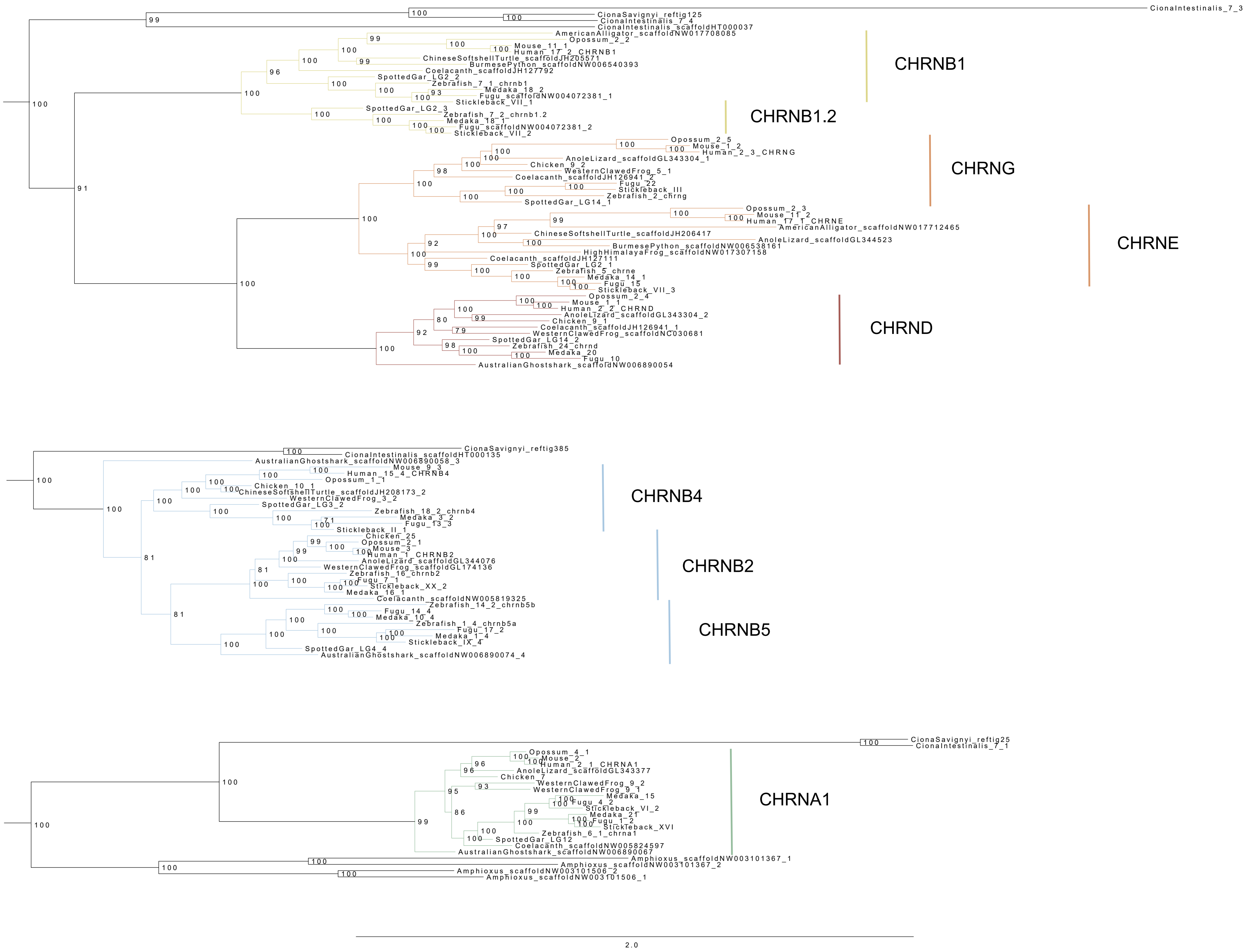

CionaIntestinalis_7_3
CionaSavignyi_reftig125
CionaIntestinalis_7_4
CionaIntestinalis_scaffoldHT000037
AmericanAlligator_scaffoldNW017708085
Opossum_2_2
Mouse_11_1
Human_17_2_CHRNB1
ChineseSoftshellTurtle_scaffoldJH205571
BurmesePython_scaffoldNW006540393
Coelacanth_scaffoldJH127792
SpottedGar_LG2_2
Zebrafish_7_1_chrnb1
Medaka_18_2
Fugu_scaffoldNW004072381_1
Stickleback_VII_1
SpottedGar_LG2_3
Zebrafish_7_2_chrnb1.2
Medaka_18_1
Fugu_scaffoldNW004072381_2
Stickleback_VII_2
CHRNB1
CHRNB1.2
Opossum_2_5
Mouse_1_2
Human_2_3_CHRNG
AnoleLizard_scaffoldGL343304_1
Chicken_9_2
WesternClawedFrog_5_1
Coelacanth_scaffoldJH126941_2
Fugu_22
Stickleback_III
Zebrafish_2_chrng
SpottedGar_LG14_1
CHRNG
Opossum_2_3
Mouse_11_2
Human_17_1_CHRNE
AmericanAlligator_scaffoldNW017712465
ChineseSoftshellTurtle_scaffoldJH206417
AnoleLizard_scaffoldGL344523
BurmesePython_scaffoldNW006538161
HighHimalayaFrog_scaffoldNW017307158
Coelacanth_scaffoldJH127111
SpottedGar_LG2_1
Zebrafish_5_chrne
Medaka_14_1
Fugu_15
Stickleback_VII_3
CHRNE
Opossum_2_4
Mouse_1_1
Human_2_2_CHRND
AnoleLizard_scaffoldGL343304_2
Chicken_9_1
Coelacanth_scaffoldJH126941_1
WesternClawedFrog_scaffoldNC030681
SpottedGar_LG14_2
Zebrafish_24_chrnd
Medaka_20
Fugu_10
AustralianGhostshark_scaffoldNW006890054
CHRND
CionaSavignyi_reftig385
CionaIntestinalis_scaffoldHT000135
AustralianGhostshark_scaffoldNW006890058_3
Mouse_9_3
Human_15_4_CHRNB4
Opossum_1_1
Chicken_10_1
ChineseSoftshellTurtle_scaffoldJH208173_2
WesternClawedFrog_3_2
SpottedGar_LG3_2
Zebrafish_18_2_chrnb4
Medaka_3_2
Fugu_13_3
Stickleback_II_1
CHRNB4
Chicken_25
Opossum_2_1
Mouse_3
Human_1_CHRNB2
AnoleLizard_scaffoldGL344076
WesternClawedFrog_scaffoldGL174136
Zebrafish_16_chrnb2
Fugu_7_1
Stickleback_XX_2
Medaka_16_1
Coelacanth_scaffoldNW005819325
CHRNB2
Zebrafish_14_2_chrnb5b
Fugu_14_4
Medaka_10_4
Zebrafish_1_4_chrnb5a
Fugu_17_2
Medaka_1_4
Stickleback_IX_4
SpottedGar_LG4_4
AustralianGhostshark_scaffoldNW006890074_4
CHRNB5
CionaSavignyi_reftig25
CionaIntestinalis_7_1
Opossum_4_1
Mouse_2
Human_2_1_CHRNA1
AnoleLizard_scaffoldGL343377
Chicken_7
WesternClawedFrog_9_2
WesternClawedFrog_9_1
Medaka_15
Fugu_4_2
Stickleback_VI_2
Medaka_21
Fugu_1_2
Stickleback_XVI
Zebrafish_6_1_chrna1
SpottedGar_LG12
Coelacanth_scaffoldNW005824597
AustralianGhostshark_scaffoldNW006890067
Amphioxus_scaffoldNW003101367_1
Amphioxus_scaffoldNW003101367_2
Amphioxus_scaffoldNW003101506_2
Amphioxus_scaffoldNW003101506_1
CHRNA1
2.0

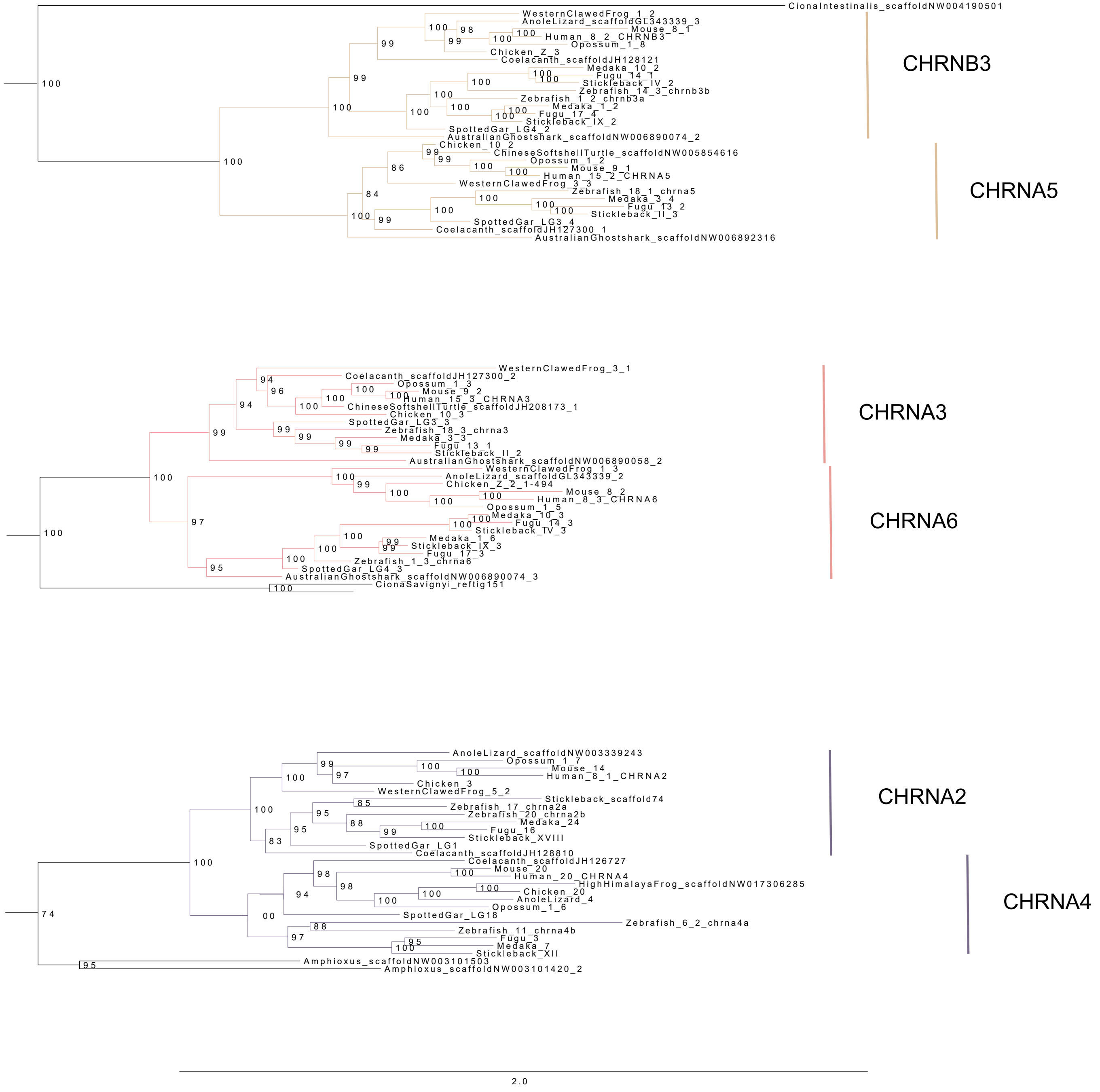
CionaIntestinalis_scaffoldNW004190501
WesternClawedFrog_1_2
AnoleLizard_scaffoldGL343339_3
Mouse_8_1
Human_8_2_CHRNB3
Opossum_1_8
Chicken_Z_3
Coelacanth_scaffoldJH128121
Medaka_10_2
Fugu_14_1
Stickleback_IV_2
Zebrafish_14_3_chrnb3b
Zebrafish_1_2_chrnb3a
Medaka_1_2
Fugu_17_4
Stickleback_IX_2
SpottedGar_LG4_2
AustralianGhostshark_scaffoldNW006890074_2
CHRNB3
Chicken_10_2
ChineseSoftshellTurtle_scaffoldNW005854616
Opossum_1_2
Mouse_9_1
Human_15_2_CHRNA5
WesternClawedFrog_3_3
Zebrafish_18_1_chrna5
Medaka_3_4
Fugu_13_2
Stickleback_II_3
SpottedGar_LG3_4
Coelacanth_scaffoldJH127300_1
AustralianGhostshark_scaffoldNW006892316
CHRNA5
WesternClawedFrog_3_1
Coelacanth_scaffoldJH127300_2
Opossum_1_3
Mouse_9_2
Human_15_3_CHRNA3
ChineseSoftshellTurtle_scaffoldJH208173_1
Chicken_10_3
SpottedGar_LG3_3
Zebrafish_18_3_chrna3
Medaka_3_3
Fugu_13_1
Stickleback_II_2
AustralianGhostshark_scaffoldNW006890058_2
CHRNA3
WesternClawedFrog_1_3
AnoleLizard_scaffoldGL343339_2
Chicken_Z_2_1-494
Mouse_8_2
Human_8_3_CHRNA6
Opossum_1_5
Medaka_10_3
Fugu_14_3
Stickleback_IV_3
Medaka_1_6
Stickleback_IX_3
Fugu_17_3
Zebrafish_1_3_chrna6
SpottedGar_LG4_3
AustralianGhostshark_scaffoldNW006890074_3
CionaSavignyi_reftig151
CHRNA6
AnoleLizard_scaffoldNW003339243
Opossum_1_7
Mouse_14
Human_8_1_CHRNA2
Chicken_3
WesternClawedFrog_5_2
Stickleback_scaffold74
Zebrafish_17_chrna2a
Zebrafish_20_chrna2b
Medaka_24
Fugu_16
Stickleback_XVIII
SpottedGar_LG1
Coelacanth_scaffoldJH128810
CHRNA2
Coelacanth_scaffoldJH126727
Mouse_20
Human_20_CHRNA4
HighHimalayaFrog_scaffoldNW017306285
Chicken_20
AnoleLizard_4
Opossum_1_6
SpottedGar_LG18
Zebrafish_6_2_chrna4a
Zebrafish_11_chrna4b
Fugu_3
Medaka_7
Stickleback_XII
Amphioxus_scaffoldNW003101503
Amphioxus_scaffoldNW003101420_2
CHRNA4
2.0
