# Supplementary material for: Evolution of vertebrate nicotinic acetylcholine receptors
Source: BMC Evol Biol. 2019 Jan 30;19:38. doi: 10.1186/s12862-018-1341-8 (PMC6354393; doi:10.1186/s12862-018-1341-8)
Supplement: Supplementary file 4 — The nAChR gene repertoire in the four teleost species zebrafish, medaka, stickleback and fugu. The zebrafish has retained 27 genes, the medaka and stickleback 28 and the fugu 29 nAChR genes. The teleost ancestor had 31 nAChR genes. (PDF 192 kb) [file 12862_2018_1341_MOESM4_ESM.pdf]

	Zebrafish	Medaka	Stickleback	Fugu
chrna7	x	x	x	x
	x	x		
chrna8	x	x	x	x
		x	x	x
chrna11	x	x	x	x
		x	x	x
chrnb2	x	x	x	x
chrnb5	x	x	x	x
	x	x		x
chrnb4	x	x	x	x
chrna5	x	x	x	x
chrnb3	x	x	x	x
	x	x	x	x
chrna3	x	x	x	x
chrna6	x	x	x	x
		x	x	x
chrna2	x	x	x	x
	x		x	
chrna4	x	x	x	x
	x			
chrna9	x	x	x	x
	x	x	x	x
chrna10	x	x	x	x
	x	x	x	x
chrna1	x	x	x	x
		x	x	x
chrne	x	x	x	x
chrng	x		x	x
chrnd	x	x		x
chrnb1	x	x	x	x
chrnb1.2	x	x	x	x
**Total**	27	28	27	28
Teleost ancestor: 31 nAChR genes				
